# Cyclin-dependent kinase inhibitors in head and neck cancer and glioblastoma—backbone or add-on in immune-oncology?

**DOI:** 10.1007/s10555-020-09940-4

**Published:** 2020-11-08

**Authors:** Christin Riess, Nina Irmscher, Inken Salewski, Daniel Strüder, Carl-Friedrich Classen, Christina Große-Thie, Christian Junghanss, Claudia Maletzki

**Affiliations:** 1 Department of Medicine, Clinic III - Hematology, Oncology and Palliative Care, Rostock University Medical Center, Rostock, Germany; 2 University Children’s and Adolescents’ Hospital, Rostock University Medical Center, Rostock, Germany; 3 Department of Oto-Rhino-Laryngology, Head and Neck Surgery “Otto Körner”, Rostock University Medical Center, Rostock, Germany

**Keywords:** CDK4/6 inhibitors, Resistance mechanisms, Predictive biomarker, Immune activation, Combination strategies

## Abstract

Cyclin-dependent kinases (CDK) control the cell cycle and play a crucial role in oncogenesis. Pharmacologic inhibition of CDK has contributed to the recent clinical approval of dual CDK4/6 inhibitors for the treatment of breast and small cell lung cancer. While the anticancer cell effects of CDK inhibitors are well-established, preclinical and early clinical studies describe additional mechanisms of action such as chemo- and radiosensitization or immune stimulation. The latter offers great potential to incorporate CDK inhibitors in immune-based treatments. However, dosing schedules and accurate timing of each combination partner need to be respected to prevent immune escape and resistance. In this review, we provide a detailed summary of CDK inhibitors in the two solid cancer types head and neck cancer and glioblastoma multiforme; it describes the molecular mechanisms of response *vs.* resistance and covers strategies to avoid resistance by the combination of immunotherapy or targeted therapy.

**Supplementary Information:**

The online version contains supplementary material available at 10.1007/s10555-020-09940-4.

## Introduction

### Cyclin-dependent kinases, inhibitors, and common alterations in head and neck squamous cell carcinoma and glioblastoma multiforme

**Fig. 1:**
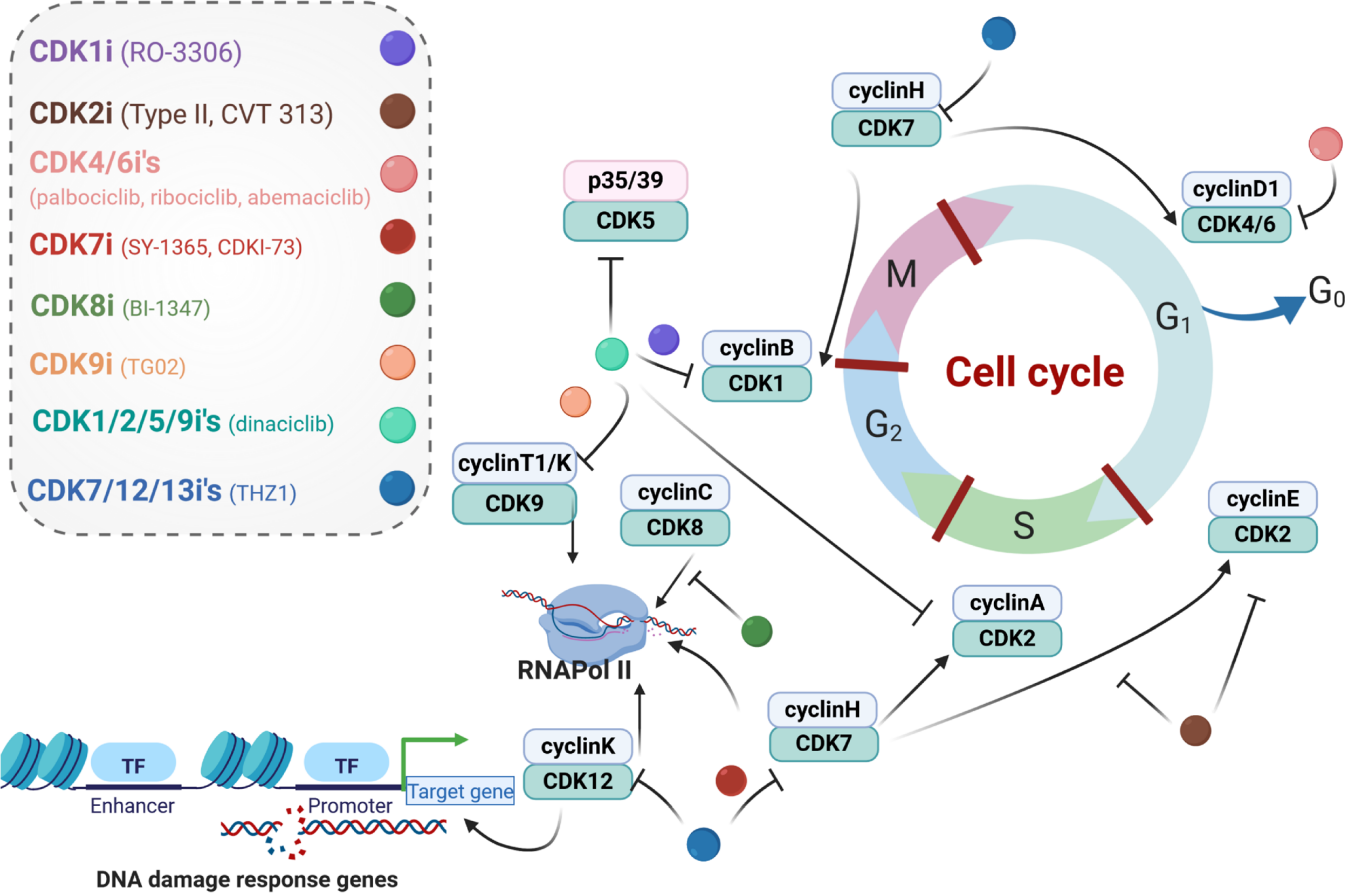
Cell cycle regulation and inhibitors targeting specific interphase CDKs. The cell cycle is tightly regulated by specific CDKs, a group of protein kinases. CDKs are constantly formed and degraded during the cell cycle. To become fully activated, a CDK binds to a cyclin protein and is being phosphorylated by another kinase. This complex also regulates downstream effects including transcription regulation, mRNA processing, and cellular differentiation

**Fig. 2:**
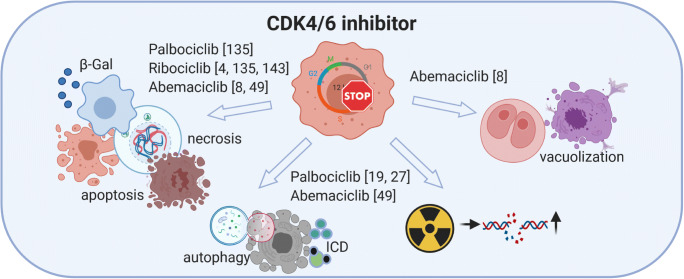
Direct effects of CDK4/6 inhibition on tumor cells. Different CDK4/6i’s inhibit cell growth and induce cell death. Despite overlapping effects, there are some characteristics unique to a specific CDKi, such as induction of vacuolization by abemaciclib. b-Gal, beta-galactosidase; ICD, immunogenic cell death. References indicate study results depicted in the figure. Created with BioRender.com

Cyclin-dependent kinases (CDK) are a family of conserved serine/threonine protein kinases. Among the 13 human CDK, some play essential roles in cell cycle regulation to ensure homeostasis and maintenance of normal cell proliferation (Fig. 1). Upon malignant transformation, these characteristics are replaced by sustained proliferative signaling and evading growth suppressors, originally formulated as hallmarks of cancer by Hanahan and Weinberg [1]. This is, among others, the result of cyclin pathway genomic alterations as recently shown on roughly 200,000 solid tumors [2]. Alterations in cyclin activating/sensitizing genes were detectable in 24% of malignancies [2]. Hence, pharmacological targeting of CDKs is in the focus of clinical research (Figs. 1 and 2).

Three CDK4/6 inhibitors (palbociclib, ribociclib, abemaciclib) are FDA-approved to treat advanced-stage or metastatic, hormone-receptor-positive, HER2-negative breast cancer (BC) (Fig. 2). Inhibitors targeting other cell cycle CDKs are currently in clinical trials, and with this progress, the spectrum of tumor targets increases. Two tumor entities with evolving relevance for CDK targeting agents include head and neck squamous cell carcinoma (HNSCC) and glioblastoma multiforme (GBM). HNSCC is the seventh common cancer worldwide with 400,000 deaths per year. Major risk factors are tobacco and alcohol consumption as well as high-risk human papillomavirus (HPV) infection. HPV-positive oropharyngeal cancer shows distinct biological characteristics and a better outcome [3]. GBM is the most common malignant primary brain tumor with a global incidence of less than 10/10,000/year, but a poor 5-year overall survival of < 5%. Pharmacological therapy options are limited for both entities, and predictive biomarkers for CDK inhibitors are yet at the beginning of identification. Still, the finding that the Cyclin D1 (*CCND1*)/CDK4/6–*CDKN2A (*p16^INK4A^)–Rb axis is altered in more than half of HNSCC and GBM cases warrants further investigations [4–7]. This will hopefully broaden treatment options for patients by applying CDKi’s in mono- or combination with chemo-/immunotherapy to prevent resistance.

## CDK inhibitors in preclinical and clinical application for HNSCC and GBM

### CDK1/2 inhibitors

CDK2 forms active complexes with cyclins E/A to promote S phase and CDK1 complexes with cyclins D/E/A/B to trigger mitosis. Agents primarily targeting these CDK’s include RO-3306 (CDK1 inhibitor) and the CDK2 inhibitors II and CVT-313.

A very recent bioinformatics approach identified CDK1, among others, as a potential response marker in HNSCC and GBM, but also as a predictor of poor outcome [8–10]. Not surprisingly, depletion of *CDK1* by siRNA causes cell cycle arrest in HNSCC cells [10]. As for CDK2, activation is linked to *CDKN2A/p16* in a way that p16 expression acts as a natural CDK2 inhibitor [11]. In 2D-cultured HNSCC cells, CDK2 knockdown enhanced radiosensitivity; however, this effect was alleviated in the 3D culture [12]. Still, comparable radiosensitizing and pro-apoptotic effects were found in CDK2-depleted GBM cells, suggesting a role for CDK2 in mediating radioresistance [13]. This CDK additionally promotes invasion by phosphorylating Rb1 (pRB) and is functionally required for GBM cell proliferation both *in vitro* and *in vivo* [13, 14]*.* Other studies also assume that CDK2 is involved in stemness maintenance and therapy resistance, making it a promising target. Bellail et al. described a mechanism by which CDK levels are maintained in GBM cells as the cell cycle progresses. In detail, they show that CDK1 interacts with the small ubiquitin-like modifier (SUMO)-specific enzyme and an ubiquitin-conjugating enzyme, which in turn intervenes with the SUMO-1-modified CDK6 and contributes to cancer development and progression. To demonstrate the interaction between CDK1 and SUMO1-CDK6, they used RO-3306 a cell-permeable, potent, and ATP-competitive Cdk1/B1 and Cdk1/A inhibitor [15]. Another study identified driver genes and key pathways within GBM cells. Genes like *AURKA, NDC80, KIF4A*, and *NUSAP1* were significantly upregulated compared to normal human glial cells [16]. To target the respective drivers, they used the pan-CDK inhibitor JNJ-7706621 with the highest potency on CDK1/2 and less potency on CDK3/4/6 and Aurora A/B. Here, JNJ-7706621 was able to reduce the proliferation, inhibit migration, and arrest cells in G2/M-phase, which ultimately led to apoptosis. Premkumar et al. investigated the effects of AZD5438, an orally bioavailable inhibitor of cyclin E-CDK2, cyclin A-CDK2, and cyclin B-CDK1 complexes, on GBM cells [17]. In Bcl-xL-silenced cells, decreased viability and reduced mitochondrial membrane potential were measured when cells were treated with increasing concentrations of AZD5438.

### CDK4/6 inhibitors

CDK4 and CDK6 inhibitors bind to the ATP cleft of the target CDK. Palbociclib is specifically active against CDK4/D1, CDK4/D3, and CDK6/D6, ribociclib inhibits the enzymatic activity of CDK4-Cyclin D1 and CDK6-Cyclin D3, and abemaciclib has higher selectivity for CDK4 than CDK6. Predictive biomarkers of response include *CCDN1* alterations as well as *CDKN2A/B/*p16^INK4A^ inactivation.

Concerning HNSCC, Patel et al. identified altered expression and activity of G1/S Cyclin A and E and CDK4/6 in 1997 [18]. Ever since CDK4/6i’s were developed and showed cytostatic activity primarily in HPV-negative cases [19]. The higher vulnerability of this subtype compared to HPV-associated HNSCC is likely because of the aforementioned *CCND1* amplification and p16^INK4A^ inactivation by gene deletion, point mutation, or transcriptional silencing *via* methylation [20]. *Vice versa*, HPV-driven cancers overexpress p16^INK4A^ and have a functionally inactivated Rb protein due to Rb degradation by the viral oncogene E7 [21]. Yet, the presence of RB1 is crucial for exerting therapeutic effects of CDK4/6i’s and constitutes an already proven biomarker in other tumor entities [22, 23]. Quite in line, *CCND1* amplification and *CDKN2A* mutations were identified as predictive biomarkers for response to abemaciclib in HPV-negative patient samples and PDX models [24, 25].

In another study, potential predictive biomarkers for ribociclib were described [26]. Using HPV-positive and HPV-negative cell lines and PDX models, cytostatic effects were restricted to the latter with functional Rb protein expression and characteristics of an epithelial phenotype. *Vice versa*, HNSCC cell lines with epithelial-to-mesenchymal transition (EMT) features, expressing mesenchymal markers vimentin and snail, were less sensitive to ribociclib [26]. The authors concluded that Rb loss could be associated with EMT and invasiveness, shown by e-cadherin and β-catenin internalization from membrane to cytoplasm as well as induced *slug* and *zeb-1* expression. The fact that EMT is associated with cancer cell resistance to anticancer drugs underscores these findings. Hence, cells with both high Rb expression and an epithelial phenotype are more likely susceptible to CDK4/6i’s.

**Fig. 3:**
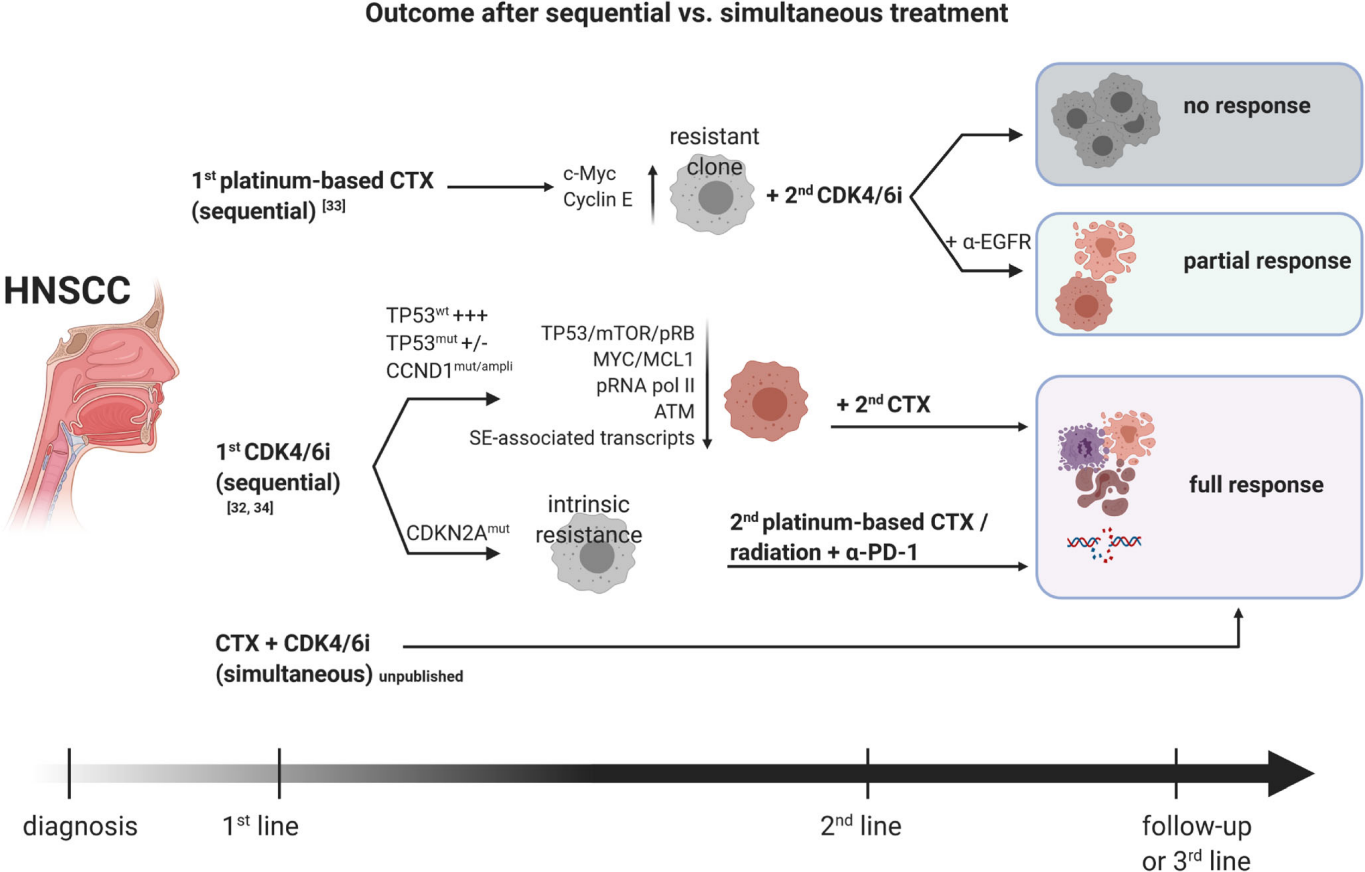
Relevance of timing on treatment response to CDK4/6i-based combination therapy. First-line therapy with platinum-based chemotherapy (CTX) triggers resistance and impedes response to CDKi (upper part). Addition of EGFR-blocking antibody cetuximab can partially suspend resistance. However, in case of first-line (middle part) or simultaneous (lower part) CDKi, developing resistance can be prevented in TP53-mutated cells, resulting in effective tumor growth control [33, 34]. ATM, ataxia telangiectasia mutated; CDK, cyclin-dependent kinase; pRNA pol II, phospho RNA polymerase II; SE-associated, super-enhancer-associated. Response includes objective response rate and complete response rate. Created with BioRender.com

Another important effect of CDK4/6 inhibition is radiosensitization, providing a chance of reducing radiation doses and related toxicity. Most HNSCC and GBM patients are treated with primary or adjuvant radiation, and some tumors (rapidly) acquire radioresistance. Göttgens et al. described effects of palbociclib only in HPV-negative HNSCC cells, dependent on p-RB1 [19]. Xie et al. additionally found a palbociclib-induced augmentation of radiotherapy by suppressing DNA double-strand break repair and inducing apoptosis in nasopharyngeal cells [27]. Palbociclib and ribociclib decrease mRNA expression level of E2F-transcriptional targets *BRCA1* and *RAD51*, interfering with radiation-induced DNA damage repair [19, 28]. For chemosensitization, the interactions appear to be more complex. Another recent study described response towards palbociclib in chemo-naive HNSCC cell lines and xenografts, but intrinsic resistance when cisplatin was given first and thus before palbociclib [29] (Fig. 3). According to this study, cisplatin-related *c-Myc* and *Cyclin E* upregulation impaired the CDKI’s effects by promoting a DNA damage-resistance phenotype. Consequently, the timing of each combination partner needs to be well-thought-out to maximize therapeutic effects and prevent resistance.

Clinically, the combination of palbociclib with anti-epidermal growth factor receptor (EGFR) monoclonal antibody cetuximab was investigated in a phase I study with recurrent/metastatic HNSCC [30]. Disease control rate was high in this initial trial (89%), with two patients showing partial response and six receiving stable disease [30]. In the phase II multicenter follow-up study, palbociclib and cetuximab were given in platinum-resistant or cetuximab-resistant HPV-negative HNSCC [31] (STable 1). Objective response was 39% in platinum-resistant and 19% in cetuximab-resistant tumors and thus similar to or even higher than reported for PD-1 inhibitors and also higher than expected in similar patients treated with single-agent cetuximab (6 months) [31]. Still, the lack of a control cohort hampers data interpretation. Just recently, results from a multicenter phase II trial were published [32]. Palbociclib was here given in combination with carboplatin for the treatment of unresectable recurrent or metastatic HNSCC (*N* = 18 patients). This combination was associated with significant treatment-related toxicity (myelosuppression) and unable to improve outcomes. Additional clinical trials involving CDK4/6i’s to treat HNSCC are ongoing and summarized in STable 1 to find out if this strategy proves successful or not*.* Our own *in vitro* data reveal the strongest growth inhibition when HPV-negative cell lines are treated simultaneously with CDKI (palbociclib) and cytostatic drugs (cisplatin, 5-FU). This regimen enhances γ-H2AX-DNA double-strand breaks and boosts apoptosis. By contrast, sequential treatment does not act synergistically (*unpublished data* and Fig. 3). This is likely because each substance interferes with the cell cycle and the impact of cell cycle inhibition may vanish in this setting. Deciphering the underlying molecular cause of resistance is in process.

For GBM, palbociclib entered clinical studies, based on preclinical data showing antitumor effects and radiosensitization [33–35]. However, two studies were terminated prematurely because of inefficacy in second-line/relapsed patients [36] (STable 2). In upcoming preclinical studies, patient-derived GBM stem cell-enriched lines also responded with transient, but not permanent cell cycle arrest [37]. Finally, the proneural GSC subtype of GBM was identified to be more palbociclib-sensitive. *In vitro* and an intracranial xenograft model, sensitivity could be attributed to αE2F1-regulated miR-17~92 [38]. Another recent study described that the combination of palbociclib with HOTAIR-EZH2 inhibitor AQB is more effective than either substance alone [39]. This combination synergistically blocked G1 phase of GBM cells *via* p-RB1 and reduced migration/invasion by Wnt/β-catenin signaling inhibition. In an intracranial xenograft model, efficient tumor growth control was reached [39].

One ongoing phase II study aims to assess the safety and efficacy of palbociclib in oligodendroglioma or recurrent oligoastrocytoma patients (STable 2). In diffuse intrinsic pontine glioma, a rare and fatal pediatric brain cancer with dysregulated G1/S cell cycle checkpoints palbociclib repressed *in vitro*/*in vivo* growth with blocking G1/S transition and other oncogenic *MYC* target genes [40]. Just like palbociclib, ribociclib was effective in preclinical pediatric CNS tumors (DIPGx7 cortical allograft) [41]. However, ribociclib was ineffective in humans because of PI3K/mTOR pathway upregulation in recurrent tumors (phase 0/1b studies) [42, 43].

Abemaciclib, as mentioned before, is structurally different from palbociclib and ribociclib. It is buried more readily to the inactive CDK4/6-ATP pocket for the smaller substituent [44]. Abemaciclib also affects other kinases including glycogen synthase kinase 3α/β and calmodulin-dependent protein kinase II α/β/γ [45], influencing the mode of cell death. While cellular senescence by G1 arrest is the primary effect, apoptosis and necroptosis were a direct consequence (Fig. 2). Recently, another type of cell death was described for abemaciclib, causing the formation of multiple swollen and dysfunctional lysosomes [46]. Lysosome-derived vacuoles containing undigested debris and remnants of organelles developed and expanded in A549-treated lung cancer cells [46]. This unique form of cell death was neither induced by palbociclib nor ribociclib and shows a very specific cytotoxic effect. Our data support this finding. Abemaciclib induced vacuolization in patient-derived GBM models, accompanied by a high abundance of LAMP1/2/Rab7a. These late endosomal markers are formed in the early stages of methuosis (Riess et al. Cell Death Discovery [47] and Fig. 2).

Raub et al. reported cell cycle arrest by p-RB1 inhibition in G1 phase and a boosted antitumor activity by adding temozolomide (TMZ) to abemaciclib *in vivo* [48]. Although overall clinical data on abemaciclib are rare for GBM, a multicenter phase I dose-escalation study included 17 GBM patients (225 patients with solid cancer in total) (STable 2). Three patients achieved stable disease, of those, two GBM patients had durable disease control [49]. Notably, both patients had *TP53* alterations, and one patient had an additional frameshift mutation in the *EGFR* gene [49]. This finding is quite intriguing, knowing that inactivating *TP53* mutations often confer intrinsic resistance towards CDK4/6i monotherapy in HNSCC and may also partially explain why abemaciclib is only rarely applied in this entity. In one study on HNSCC cell lines and tumor xenografts, abemaciclib affected tumor growth and inhibited activation of AKT and ERK, but not mTOR [25]. While the latter was identified as a potential target, the authors applied combinations with everolimus and described a cooperative antitumoral effect *in vivo* [25]*.* Finally, the potential of abemaciclib in treating HNSCC was preclinically proven on a large series of patient-derived xenograft models [24]. Treatment response positively correlated with *CCND1* and *CDKN2A* genomic alterations.

### CDK7/8/9/12/13 inhibitors

CDKs regulating gene expression apart from cell cycle control constitute additional therapeutic targets. Specifically, the CDKs 7/8/9/12/13 are involved in RNA polymerase II transcription regulation [50–52]. Though the complexity exceeds the scope of this review, we included some information gathered from HNSCC and GBM studies inhibiting these CDK’s.

An ongoing phase I trial investigates safety and antitumor activity of selective CDK7i SY-1365 in patients with advanced solid tumors (*NCT03134638*). Based on preclinical data, SY-1365 mediated growth inhibition on HNSCC and GBM cells at nanomolar concentrations [53]. Molecularly, the pro-survival protein MCL1, a member of the B cell lymphoma-2 (Bcl) family that is overexpressed in ~ 90% of HNSCC cases, was significantly downregulated by this substance, and cancer cells with low BCL-xL expression were more sensitive to SY-1365 [54]. The more global acting CDK7/12/13i THZ1 induced apoptosis, inhibited tumor growth, and prevented resistance in murine xenograft models [53]. In nasopharyngeal cancer cells, THZ1 specifically suppressed super-enhancer-associated transcripts [55], a class of regulatory regions often enriched in *bona fide* oncogenes [56]. THZ1 specifically reduced RNA polymerase II bindings to interfere with cell proliferation, apoptosis, and migration. In conjunction with BRD4 inhibitor (JQ1), THZ1 impaired cell proliferation, induced apoptosis and senescence, and recapitulated by dual BRD4 and CDK7 knockdown [57]. Apoptosis induction could be traced back to reduced H3K27ac enrichment in the super-enhancer region of yes-associated protein 1, a transcriptional coactivator [57].

In GBM, one study reported reduced U87 cell proliferation and increased cleaved caspase-3 levels after THZ1 application *in vitro* and *in vivo* [58]. Global downregulation of super-enhancer-related genes accompanied therapeutic effects. Finally, the authors identified CDK7 as a prognostic marker for both lower-grade gliomas and GBM [58].

**Fig. 4:**
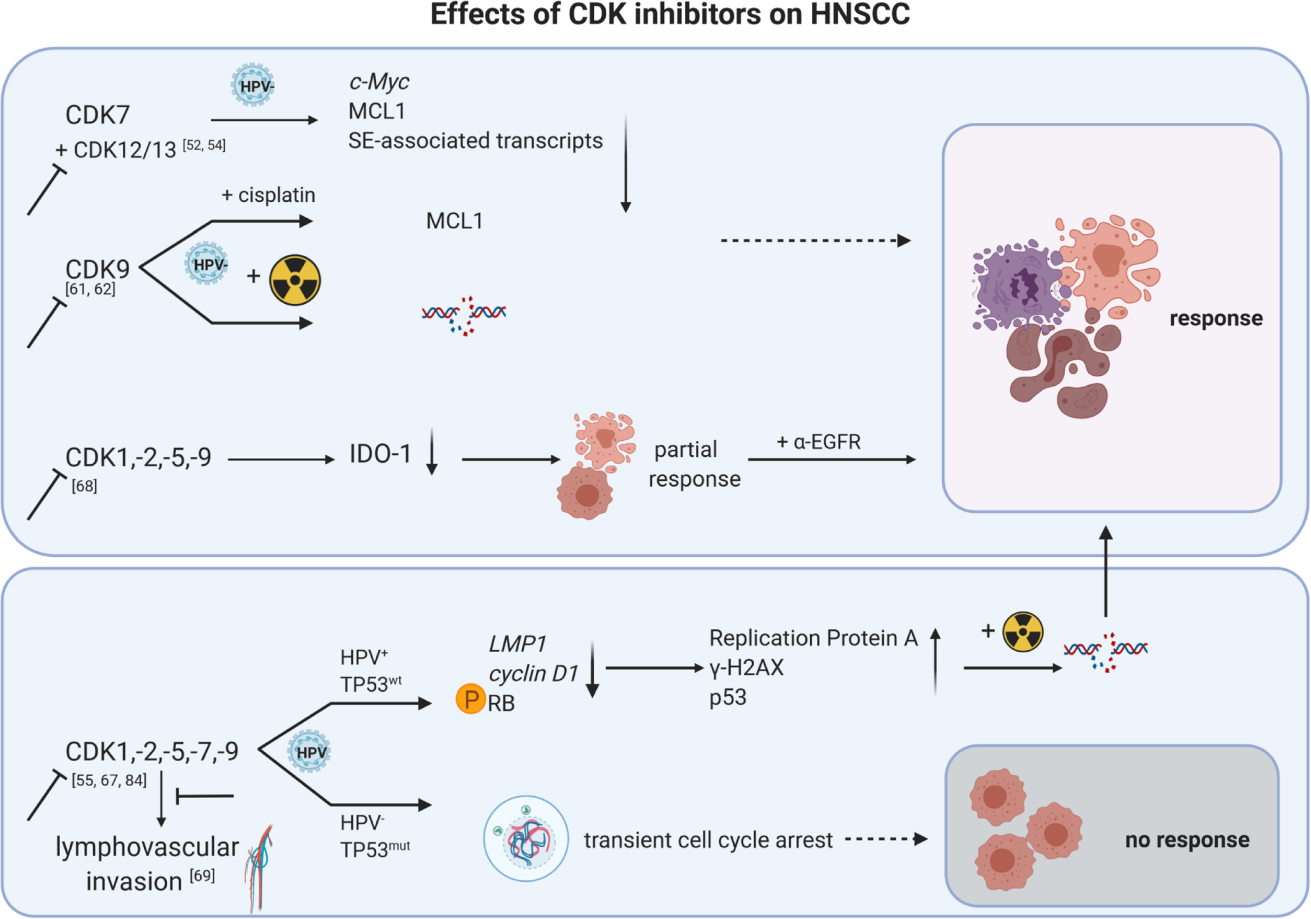
Effects of CDK inhibitors on HNSCC. Dependent on the target, single or global CDKi alone and in combination with chemo- or radiotherapy increases effects of the monotherapy to control tumor growth. In most cases, HPV-negative/TP53^mut^ cases respond better than HPV-positive/TP53^wt^ tumors. Pan-CDK1/2/5/7/9i inhibition is an exception in HPV-related cancer [67]. Pan-CDKi induces DNA damage followed by p53-dependent cell death in HPV-positive, but not HPV-negative HNSCC. HPV-positive cells respond with accumulation of replication protein A complex accompanied by increases in γ-H2AX levels and apoptosis. SE-associated, super-enhancer-associated; IDO1, indoleamine 2,3-dioxygenase. Response includes objective response rate and complete response rate. Created with BioRender.com

**Fig. 5:**
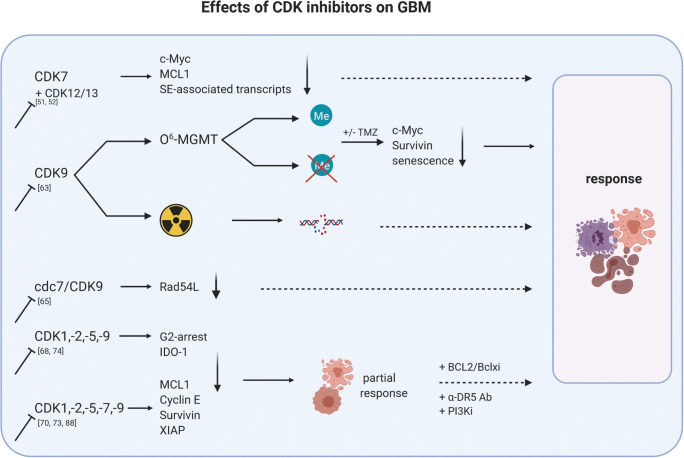
Effects of CDK inhibitors on GBM. Dependent on the target, single or global CDKi alone and in combination with chemo- or radiotherapy increases effects of the monotherapy to control tumor growth. Generally, effects of the monotherapy are increased by applying specific anti-apoptosis or PI3K inhibitors [70–73]. Of note, CDK9i is effective in both MGMT-methylated and unmethylated GBM [63], while global CDKi interferes with IDO1 to reduce immunosuppressive activity [68]. SE-associated, super-enhancer-associated; MGMT, O(6)-methylguanine-DNA methyltransferase; ME, methylated; XIAP, X-linked inhibitor of apoptosis protein. Response includes objective response rate and complete response rate. Created with BioRender.com

Only a few studies investigated CDK9 as a treatment strategy for HNSCC and GBM. CDK9 allies with T-type cyclins and Cyclin K and is strictly regulated at different levels [59]. CDKI-73, a potent CDK9i, induced apoptosis as a single agent and acts synergistically with cisplatin through *MCL1* downregulation in HNSCC [60]. Storch et al. described radioresistance in HNSCC overexpressing CDK9 and restored radiosensitivity after CDK9 knockdown accompanied by Rb hypophosphorylation and decreased Cyclin D1 level [61] (Fig. 4). Moreover, pharmacological inhibition with the global CDKI ZK304709 did not induce radiosensitivity, likely because of only partial CDK9 inhibition at the applied doses [61]. Another CDK9-targeting agent is TG02. This substance showed activity in primary patient-derived GBM cell lines and in intracranial xenograft models *via* transcriptional inhibition of anti-apoptotic proteins, including MCL1 and survivin [62, 63]. TG02 additionally boosted TMZ effects by impairing glycolysis and mitochondrial function [62]. The activity was independent of O^6^-methylguanine DNA methyltransferase expression and no resistance observed, even after prolonged treatment. The combination of TG02 and TMZ led to an accumulation of MCL-1, loss of c-MYC, and senescence [63] (Fig. 5). PHA-767491, a selective ATP-competitive dual inhibitor cdc7/CDK9, was described to enhance replicative stress, apoptosis, and radiosensitivity in GBM *via* suppression of RAD54L *in vitro* and *in vivo* [64] (Fig. 5). Another very recent study describes sensitization to TG02 by IFN-β. Pre-exposure to IFN-β augmented cell death by suppressing phosphorylation of the CDK9 target RNA polymerase II and thus inhibiting DNA-dependent mRNA synthesis [65].

Currently, CDK9-targeting is investigated as a second-line agent in a phase I trial metastatic or progressive solid tumors and also in recurrent and in newly diagnosed IDH1R132H-non-mutant anaplastic astrocytoma or GBM (STable 2).

CDK8 is another target yet to be tested. CDK8 acts as an oncogene and mediates aberrant Wnt/β-catenin pathway activation. This pathway controls stem and progenitor cell proliferation, survival, and cell differentiation and is aberrantly activated in GBM and other malignancies [71, 72]. The only prior study in HNSCC revealed a strong association of CDK8 overexpression with lymph node metastasis and advanced clinical stages [73]. Hence, targeting CDKs on a more global level than 4/6 is promising for these cancers.

### Other multi- and pan-CDK inhibitors

Flavopiridol, a synthetic flavone was the first CDKI entering the clinic [74]. In most cases, the use proved difficult in the clinical setting, with a complex pharmacokinetic profile, a range of side effects, and an unknown mechanism of action [75, 76].

Seliciclib is a second-generation CDK1/2/5/7/9i that competes for ATP binding sites on these CDKs. Already in 2002, antitumoral and pro-apoptotic effects were described on HNSCC cells [77]. Later on, patients with treatment-naïve locally advanced nasopharyngeal carcinoma received seliciclib at high or low dose twice daily. All patients (*N* = 20) achieved stable disease and a 50% reduction of lymph node masses. Effects were associated with increased apoptosis, necrosis, and decreased plasma EBV DNA [78]. This latter is of high relevance, given the known association of EBV infection and latency with malignant transformation [79]. Pre- and post-treatment gene expression analysis revealed transcriptional upregulation of *LMP1* and reduced MCL1, Cyclin D1, and p-RB1 protein levels (Fig. 4) [54, 60]. Similar to other CDKI, seliciclib is more potent against *TP53* wild-type than *TP53*-mutant cells [78]. In a preclinical follow-up study, significant DNA damage followed by p53-dependent cell death was described in HPV-positive, but not HPV-negative HNSCC [80]. HPV-positive cells responded with an accumulation of replication protein A complex that binds to single-stranded DNA accompanied by slight rises in γ-H2AX levels [80]. Therefore, HPV positivity was proposed as a biomarker for seliciclib sensitivity. These finding surprises as HPV-positive HNSCC are usually resistant to other CDKI. Results were partially confirmed thereafter, showing higher levels of apoptosis in HPV-positive *vs.* HPV-negative cells [66]. Seliciclib also enhanced radiation-induced G2 arrest in HPV-negative cells *via* inhibition of homologous recombination (HR). HR is one main double-strand break repair pathway, and seliciclib inhibits several DNA repair proteins (Exo1, BRCA1, and CtIp). HPV-positive cell lines were not sensitive to HR inhibition but could be radiosensitized by PARP-1i olaparib. In contrast, the authors only achieved radiosensitization by olaparib when seliciclib was added to HPV-negative cell lines [66].

In GBM, seliciclib and its next-generation derivative CYC065 (high-affinity for CDK2/5/9) restored apoptosis in human GBM cells and neurospheres. Seliciclib induced TRAIL-mediated apoptosis and suppressed survivin, XIAP, MCL1, and Cyclin E [67–69, 81]. Effects improved in combination with the human death receptor 5 antibody drozitumab or TMZ [67, 82] (Fig. 5). One study also described synergy with PI3Ki [70] (Fig. 5).

AT7519 (targeting CDK1/2/4/5/6/9) is another compound in early clinical development. AT7519 has favorable pharmacokinetic characteristics compared to seliciclib, including increased serum half-life (3 h *vs.* ~ 1 h) and delayed metabolic deactivation (described as metabolic switching from the major carbinol oxidation pathway) [83, 84]. Antiproliferative, pro-apoptotic, and chemosensitizing effects in nasopharyngeal carcinomas were described alone and in combination with cisplatin [85]. Mechanistically, AT7519 decreased p-RB1 and CDK2 activity. Of note, AT7519 likewise reduced phosphorylation of RNA polymerase II *via* CDK1/9 and affected expression levels of the oncoprotein *N*-*Myc*, making this compound a candidate for treatment of *N*-*Myc*-driven cancers. Zhang et al. recently identified AT7519 for controlling lymphovascular invasion in HNSCC patients [86]. Clinical trials are on the way to prove efficacy in HNSCC and other malignancies (STable 1).

Syn et al. showed that the pan-CDKI roniciclib (targeting CDK1/2/3/4/7/9) has anti-neoplastic activity as a single agent and potentiates cisplatin lethality in preclinical nasopharyngeal carcinoma models [87]. Roniciclib restricted tumor growth comparable to low-dose cisplatin. The combination of both was well tolerated *in vivo* and had synergistic efficacy [87]. Initial clinical trials on roniciclib principally proved safe application, with gastrointestinal toxicities comparable to abemaciclib [87].

Finally, dinaciclib, another experimental CDK1/2/5/9i, has been rarely explored in HNSCC and GBM. Dual treatment of dinaciclib together with Bcl-2 and Bcl-xLi ABT-737 induced apoptotic cell death in GBM, because of proteasomal degradation of Mcl-1 [88]. In a subsequent study, dinaciclib (but not other CDKi’s) exerted high toxicity against Bcl-xL-silenced cells. Morphological effects included cell shrinkage, mitochondrial dysfunction, DNA damage, and reinforced phosphatidylserine externalization [17]. Mechanistically, the mitochondrial membrane potential was disrupted leading to cytochrome c, AIF, and smac/DIABLO release into the cytoplasm. Cyclin D1, D3, B1, total-RB1, and BAX/BAK levels increased. Furthermore, proteolysis of DNA repair proteins RAD51/Ku80 augmented apoptosis [17]. Our preliminary data reveal strong pro-apoptotic and necrotic effects in 2D and 3D *in vitro* HNSCC and GBM models, accompanied by gross molecular alterations (*unpublished data*) that are promising. However, several phase I/II trials reported immune-related adverse effects such as neutropenia and leukopenia. These have to be managed before carrying on.

## Blood-brain barrier penetration of CDK inhibitors

The blood-brain barrier (BBB) consists of specialized endothelial cells with tight junctions and transport proteins that serve to restrict brain uptake of drugs, including systemic chemotherapies [89]. Hence, effective BBB permeability is a prerequisite for the successful application of therapeutic drugs in patients with brain cancer or brain metastases. Current knowledge on BBB penetration of CDKi’s is summarized in Table 1.

**Table 1:** Reported effects of CDKi’s on the immune system and blood-brain barrier (BBB) penetration

CDKI [reference]	Target	Immunomodulatory effects	BBB penetration
Palbociclib [93–95]	CDK4/6	- Boosted antitumor immunity by enhanced antigen presentation - Enhanced sensitivity of immune-refractory tumor cells - Induced stromal senescence - Reduced T and increased granulocytic MDSC infiltration	Yes
Ribociclib [94, 95]	CDK4/6	- PD-L1 upregulation independently of RB status	Yes
Abemaciclib [93, 96]	CDK4/6	- Boosted antitumor immunity by enhanced antigen presentation - MHC class I/II upregulation - Selective suppression of regulatory T cells	Yes
Dinaciclib [68, 97–99]	CDK1/2/5/9	- *Bona fide* immunogenic cell death-inducing agent - Induces expression of type I IFN response genes and *damage-associated molecular patterns* expression in tumor cells - Suppresses activation of IFNγ-induced IDO-1 upregulation in patient-derived GBM cells - Mediated infiltration of NK cells and macrophages	Not reported
THZ1 [100–102]	CDK7/12/13	- Downregulates PD-L1 expression by inhibiting MYC activity - Is more effective in the presence of PBMCs - Coculturing of PBMC with THZ1-pretreated cells increases IFNγ production - Enhances efficacy of α-PD-1 therapy by recruiting CD8^+^ T cells in NSCLC	Yes
YKL-5-124 [103]	CDK7	- Improves immune responses - Enhances IFNγ signaling, TNF-α, and chemokine ligand 9/10 release	Not reported
CDKI-73 [61]	CDK9, eukaryotic translation initiation factor 4E (eIF4E)	- Modulates the secretion of pro-inflammatory cytokines	Not reported
TG02 [63, 66]	CDK9	- Not reported	Yes
PHA-767491 [104]	Cdc7/CDK9	- Suppresses T cell activation, antigen-driven proliferation, and effector functions *in vitro* - In combination with α-PD-1 boosted effects of the monotherapy	Not reported
Flavopiridol [76]	CDK 1/2/4/6/7/9, GSK3β, Cdc2	- Increase neutrophils’ apoptosis	Yes
Seliciclib [67, 105]	CDK1/2/5/7/9	- Not reported	Yes, but limited
AT7519 [69]	CDK1/2/4/5/6/9	- Induces neutrophil apoptosis to promote inflammation resolution in preclinical models of lung inflammation - Induces human eosinophil apoptosis	Not reported
SPH3643 [106]	CDK4/6	- Not reported	Yes
ON123300 [107]	Ark5, CDK4, β-type platelet-derived growth factor receptor	- Not reported	Yes

Though all CDK4/6i’s have the capability of penetrating BBB, differences in efficacy may exist, and Raub et al. indeed described better BBB penetration of abemaciclib compared to palbociclib [48]. Mechanistically, the efflux transporters P-glycoprotein and BC-resistance protein may restrict the BBB penetration of palbociclib, which reduces effectiveness [106, 107]. One study described ameliorated palbociclib efflux and increased drug concentrations in the brain by adding mTOR inhibitors [108].

Experimental prove of BBB penetration by ribociclib was given in preclinical pediatric central nervous system tumors (DIPGx7 cortical allograft) [41]. Still and just like palbociclib, efflux transporter ABCB1 limited BBB penetration, a mechanism not yet described for abemaciclib. Hence, co-administration of ABCB1-inhibitors like elacridar is promising for future research to increase efficacy [109].

Yin et al. developed a series of CDK4/6i’s and identified a substance termed “compound 11” with the potential to inhibit CDK4/Cyclin D1 and CDK6/Cyclin D3 activity as well suppressing tumor p-RB1 at doses far below the FDA-approved CDKi’s [110]. Using an orthotopic GBM model, “compound 11” prolonged the life span of mice. The authors explained the high efficacy with free-crossing of the BBB to reach levels in the brain that effectively inhibited CDK4/6 activity (total brain/plasma ratio: 4.1 *vs.* abemaciclib: 0.21) [110]. Another group also designed a new brain-penetrating CDK4/6i with potency as effective as abemaciclib in Rb-wt cells [111]. This molecule had low molecular weight and topological polar surface area but no active efflux [111]. Likewise, SPH3643 a novel orally active small CDK4/6i effectively penetrated the murine cerebrum and significantly decreased GBM growth [104].

In 2014, the multikinase inhibitor ON123300 was identified as a potent agent for brain tumor chemotherapy [105]. ON123300 was a strong inhibitor of Ark5 and CDK4, as well as β-type platelet-derived growth factor receptor. Single-agent application caused a dose-dependent suppression of phosphorylation of Akt as well as activation of Erk in brain tumors [105]. The missing follow-up information, however, raises questions on efficacy.

Another BBB penetrating agent with antitumoral activity in GBM is THZ1. Preclinical *in vivo* studies revealed highest THZ1 brain parenchymal concentrations following intravenous dosing [97]. Also, TG02 passes the BBB and had activity in patient-derived and long-term GBM cells, in glioma-initiating cell lines, and a syngeneic orthotopic GBM model [62, 65].

Regarding BBB penetration of multi- and pan-CDKi’s, different results are reported in literature (please see [112] for details). Seliciclib, for instance, demonstrated only limited brain exposure in rats because of P-glycoprotein [103] and thus similar mechanisms as described before.

## Immune modulatory effects: increased or suppressed immunity upon CDK inhibition?

HNSCC and GBM have an immunosuppressive microenvironment characterized by secretion of immunosuppressive cytokines (TGF-β, IL-10), loss or downregulation of MHC class-I and antigen processing machinery components, upregulation of immune-checkpoint molecules, dysfunctional T and natural killer (NK) cells, as well as recruitment of immunological suppressors, including regulatory T cells (Treg), myeloid-derived suppressor cells (MDSC), and tumor-associated macrophages (TAM). Still, both entities differ in terms of immunogenicity. HNSCC are hypermutated and often harbor an “IFNγ-signature”, i.e., they are highly infiltrated with immune cells. In HPV-positive cases, the number of tumor-infiltrating CD8^+^ T cells is associated with better survival compared to their HPV-negative counterpart [113–116]. GBM is quite different. Despite rare metastasis outside the brain, tumors are very immunosuppressive with numbers of immunosuppressive cells being equal to or even exceeding those of tumor cells [117]. Tumor-associated astrocytes play an important role in augmenting GBM malignancy and fostering immune evasion through PD-L1 and STAT3 upregulation, IL10 secretion, and overexpression of growth/differentiation factor 15 [118, 119].

Coping with these strategies constitutes a challenge for immunotherapeutic approaches and raises the question of whether CDK inhibition improves or impairs immune function. Cyclins and CDKs play crucial roles in development, differentiation, and immune cell activation. Petroni et al. recently reviewed the complex interaction of CDKi’s with cancer cells and normal (tumor-infiltrating immune) cells [120]. It is now clear that CDKi’s modulate the immune system [121]. These include, among others, ICAM1-mediated NK cell engagement and Treg/MDSC reduction upon RB pathway activation, finally enhancing sensitivity to immunotherapy [90, 122]. Hence, CDK inhibition in HNSCC and GBM may have the potential to break immune tolerance and thus improve outcomes. Still, the so far limited information available in these two entities led us to discuss recent data from other entities.

**Fig. 6:**
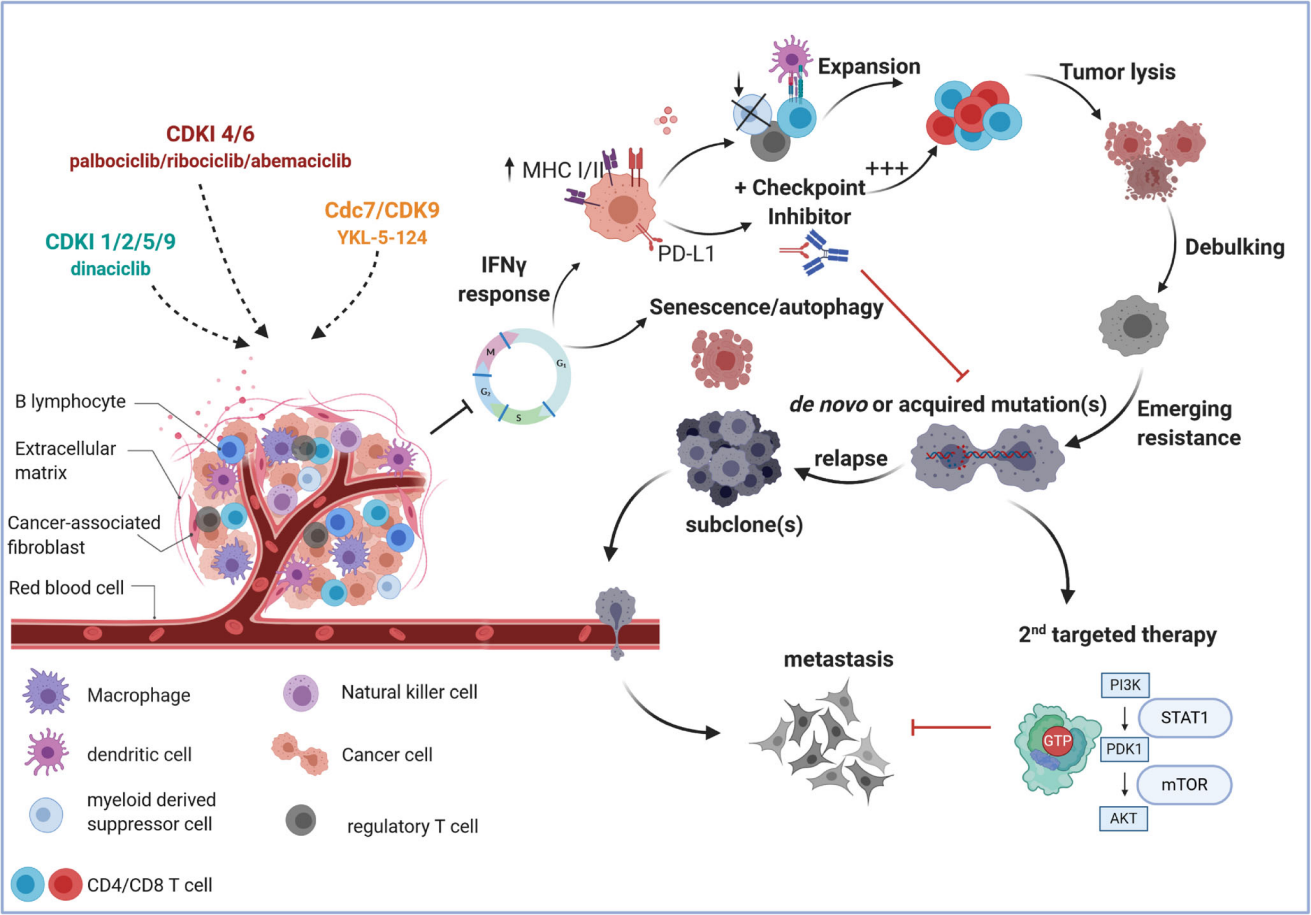
Immune modulatory effects of CDKi and strategies to improve outcome. CDKi treatment induces an IFNγ response, accompanied by MHC I/II upregulation but also stabilization of PD-L1 on the tumor cells’ surface. Addition of immune-checkpoint inhibitors may prevent resistance. This in turn suppresses regulatory T cells and induces cytotoxic T cell expansion, leading to efficient tumor cell killing and clinical response. Because of emerging resistance, either by selecting rare tumor cells with preexisting mutations (*de novo*) or selecting cells with new mutations (acquired), patients frequently relapse. To prevent metastasis, molecular targeted therapy is indicated. Most resistant tumor cells activate or upregulate specific pathways; hence, resistance may in this case present a specific target for PI3K/mTOR and MET/TRK inhibitors. Created with BioRender.com

First reports on the interference with the immune system date back to 2012, when flavopiridol was shown to increase neutrophils’ apoptosis *via* declined levels of the anti-apoptotic protein Mcl-1, while Bcl2A was unaffected [102]. Later in 2017, Goel et al. identified another mode of action beyond cell cycle arrest. By performing genome-wide transcriptome analysis of human and murine mammary cancer specimens, boosted antitumor immunity after abemaciclib and palbociclib were confirmed on serial biopsies from a clinical BC trial [90]*.* Both CDKi’s enhanced antigen presentation because of the re-expression of endogenous retroviral elements and reduced activity of DNA methyltransferase 1. This, in turn, suppressed Treg proliferation and stimulated cytotoxic T cells [90] (Fig. 6). Comparable effects were seen in RB1^+^ Ewing sarcoma; cells responded with a prototypical IFN response upon abemaciclib treatment, including higher gene transcripts of *STAT-1, IRF-1, CXCL-10, IDO-1,* and *HLA-B* [123]. Abemaciclib stimulated a T cell-inflamed microenvironment because of MHC class I/II upregulation on tumor cells and improved NFAT (nuclear factor of activated T cells) signaling in tumor-infiltrating lymphocytes to overcome defective T cell receptor signaling [93]. Notably, abemaciclib selectively suppresses Treg proliferation through repression of DNA methyltransferase 1 expression. The effect was specific for Tregs and did not affect other T cells [123]. The preserved T cells’ ability to respond to proliferative signals is intriguing because cell cycle control is alike in healthy and malignant cells. CDKs and their inhibitors can support clonal T cell expansion [124] (Fig. 6), reflected by stronger T cell infiltration in several CDKi studies. Lately, the neoMONARCH trial confirmed clinical responses to abemaciclib (plus anastrozole) in early-stage HR^+^/HER2^−^ BC patients and found upregulation of inflammatory and T cell-related pathways (assessed by RNA-seq) [125]. However, a Ki67 rebound was seen after the treatment, indicating the need for long-term CDKi treatment. The Ki67 rebound was also found for palbociclib in a neoadjuvant setting [125]. Long-term treatment is most likely feasible for abemaciclib as other CDK4/6i’s exhibit distinct side effects [4].

Another immune interaction was found for palbociclib by enhancing the sensitivity of immune-refractory cancer cells after prior CTL-mediated immunotherapy [126]. Cancer cells escape immune-mediated apoptosis by upregulation of *synaptonemal complex protein 3* (*SCP3*) and the stemness factor *NANOG*. Using a SCP3^high^ immune-refractory BC-xenograft model, palbociclib inhibited tumor growth and prolonged survival [126]. In a melanoma study, palbociclib-induced stromal senescence triggered a senescence-secretory phenotype in cancer cells (IL6, IL8, CXCL-1, VEGF), fostering growth *in vivo via* reduced T and increased granulocytic MDSC infiltration [127]. Supported by findings in *Pten*-null prostate cancers [128], stromal cells should be protected from senescence to avoid counterbalance of the tumor microenvironment (TME). While direct short- and long-term effects of CDK4/6i’s on the cell cycle vanish, an experimental study on ductal BC recently uncovered that genes associated with differentiation, inflammation, IFNγ response, and antigen processing are preserved in tumor and TME after palbociclib treatment [129].

Dinaciclib is a *bona fide* immunogenic cell death-inducing agent. It induces the expression of type I IFN response genes and *damage-associated molecular patterns* expression in tumor cells, which upsurges tumor antigen processing and presentation (Fig. 6). Our group identified that dinaciclib suppresses activation of IFNγ-induced *IDO-1* upregulation in patient-derived GBM cells (Fig. 5) [47]. While *IDO-1* upregulation is a common acquired resistance mechanism with global immunosuppressive effects, our findings support the incorporation of CDKi in immunotherapeutic concepts [94]. Dinaciclib-mediated infiltration of NK cells and macrophages was recently reported in A549 xenografts. Immune infiltration was boosted *via* TRAIL delivered by extracellular vesicles [95]. Another interesting approach is based on vaccination with dinaciclib-killed immunogenic tumor cells. After reimplantation, tumor growth was prevented in murine syngeneic tumor models, antitumor activity augmented by an α-PD1 antibody [96].

Recently, a nano-immunotherapy approach targeting tumor-associated myeloid cells (TAMC) in GBM was developed [130]. This α-PD-L1 antibody-coupled lipid nanoparticle with encapsulated dinaciclib extended mice overall survival and decreased TAMC [130]. Notably, also human TAMCs were effectively targeted [130].

Although these data provide robust evidence for the interference of certain CDKi with the immune system, the immunomodulatory potential in HNSCC and GBM remains unexplored. Likewise, little is known about other CDKi’s. The dual Cdc7/CDK9i PHA-767491 potently suppressed T cell activation, antigen-driven proliferation, and effector functions *in vitro* [101]. Specifically, the Cdc7/CDK9 blockade inhibited Erk phosphorylation in different T cell populations, suppressed TNF/IFNγ-cytokine release, and induced caspase-3-dependent NF-κB p105 degradation [101]. Hence, these kinases may be involved in signal transduction downstream of the T cell receptor. However, the clinical significance of these tolerogenic effects remains unclear and has to be addressed prospectively.

In contrast, specific CDK7/9 targeting of tumor cells improves immune responses and acts synergistic with α-PD1 antibodies [98, 99]. Zhang et al. confirmed IFNγ signaling, TNF-α, and chemokine ligand 9/10 release in YKL-5-124 (CDK7/12/13i)-treated lung cancer [100]. Upon treatment with supernatant from responding cells, ovalbumin-specific OT-I T cells were stimulated to express CD69, TNFα, and IFNγ. Therapeutic application *in vivo* (schedule: q5dx5, ip) controlled tumor growth and activated CD4^+^ T cells [100]. The combined application of YKL-5-124 and α-PD-1 boosted the effects of the monotherapy in multiple aggressive tumor models [100]. The authors also proposed a new mechanism. Induction of genomic instability may contribute to secretion of pro-inflammatory cytokines/chemokines. A highly selective CDK9i (MC180295) even reactivated tumor-suppressor genes by chromatin remodeling without DNA methylation effects. The cellular response sensitized tumor cells to α-PD-1 antibodies [131]. This synergistic activity of combined CDKi- and immune-checkpoint-inhibition therapy is due to CDKi-dependent PD1/PD-L1 axis activation [132] (Fig. 6). Even though immune evasion may be higher, specific molecular targets are created (Fig. 6). For example, palbociclib- or ribociclib-mediated tumor immune evasion and resistance could be abrogated by α-PD-1 to restore tumor-infiltrating lymphocytes and therapeutic efficacy [91, 92]. Hence, immune-checkpoint-blocking antibodies improve the therapeutic effects of CDKi in murine tumor models [93, 133, 134]. The clinical significance of the combination is under investigation.

Combining CDKi’s with other targeted therapies provides further options to improve the immune response (Fig. 6). PI3Kα and CDK4/6i’s elicited cell cycle arrest, apoptosis, and calreticulin exposure and enhanced tumor immunogenicity in a syngeneic triple-negative BC model [135]. Antigen presentation (*HLA-A, HLA-DMA, CTSD, ICAM, RELB, PSME1*, *TAPBP)* and CD86 expression increased upon combination therapy [135]. *In vivo*, combined PI3Kα/CDK4/6 inhibition augmented T cell and NK cell infiltration and PD-1/CTLA-4 co-expression. The creation of an inflammatory microenvironment was accompanied by reduced immunosuppressive monocytic MDSC and decreased Treg proliferation [135].

It is tempting to speculate that CDKi’s have the potential to become the backbone for immune-checkpoint-blocking therapies. Still, immunoevasion downstream of cell cycle blockade and tolerogenic effects may occur. Therefore, research has to focus on dosing schedules and accurate timing of each combination partner to circumvent immunoevasion.

## Resistance mechanisms and novel combination approaches

Cancer is a consistently developing multicellular ecosystem [136]. Targeted therapy resistance is a common challenge in the clinic. Research, mainly for palbociclib, revealed that despite promising initial responses, resistance emerges in virtually all patients [137]. Mechanistically, genetic and phenotypic heterogeneity is one primary reason for resistant clones under selection pressure [138]. Resistance is a result of selecting rare tumor cells with preexisting mutations (*de novo*) or selecting cells with new mutations (acquired).

So far, limited information is available on resistance mechanisms in HNSCC and GBM. HPV positivity itself constitutes an intrinsic resistance mechanism to CDK4/6i’s; acquired resistance may involve alterations in CDK2/6, Cyclin E, p21, p27, Rb, the PI3K-mTOR pathway, and the fibroblast growth factor receptor [139]. Additional acquired resistance mechanisms, mainly identified in BC, involve decreased dependence on estrogen receptor (ER) signaling (due to ER-alpha mutations), ER downregulation, IL6/STAT3 alteration, and DNA damage response pathways *in vitro* [137, 140]. Notably, these resistance mechanisms were detected in clinical biopsies from BC patients progressing upon palbociclib [137]. Targeting IL6/STAT3 activity and DNA repair deficiency using a specific STAT3i combined with a PARPi decreased acquired palbociclib resistance [137], and a clinical trial is currently assessing tolerability of on oral STAT3i (clinicaltrial.gov identifier *NCT03195699*).

*De novo RB1* mutations correlate with acquired resistance to palbociclib or ribociclib in metastatic BC patients [23]. Next-generation sequencing from circulating tumor DNA (ctDNA) after treatment identified multiple somatic *RB1* mutations that were not detected in pre-treatment ctDNA analysis [23]. Mutations affected different exons: a frameshift encoding for a truncated protein (exon 8), a deleterious missense variant (exon 16), and an in-frame exon 22 deletion. These changes lead to exon 22–24 skipping and loss of a binding region for the E2F-based transcription factor. Previously, loss of E2F-based transcription factor binding regions was described in lung cancer only [141]. The somatic *RB1* mutations in three metastatic BC patients showed a rapid dynamic, and the authors concluded a selection under the pressure of CDK4/6i rather than being spatially subclonal. This was the first report on resistance because of *de novo RB1* mutations in patients. This clinical experience confirmed experimental data on *RB1* loss or *CCNE1* amplification in T47D cells upon CDKi [22]. Using a CDK4/6-sensitive PDX model, acquired resistance was induced *via* long-term *in vivo* ribociclib treatment. Here, a subclonal *RB1* frameshift mutation (RB1 p.M695fs*26) was identified in resistant tumors upon CDK4/6 inhibition. To prevent CDK4/6 resistance, CDK4/6i, and PI3Ki, combinations were applied to treatment-naïve tumors *in vivo* [142]*.* While this upfront combination prevented the development of CDK4/6 resistance, it could not resensitize cancers with acquired resistance. The rationale for combining PI3K pathway inhibitors with CDKi is based on the response to long-term CDK4/6i treatment because cells use PI3K-dependent upregulation of *Cyclin D1* along with CDK2-dependent p-RB1 [22]. In GBM, combinations of abemaciclib and c-Met/VEGFR2/TIE2/Trk-i altiratinib were able to overcome resistance [143].

Quite in line, mTORC1/2i collaborates with palbociclib inhibiting E2F function in ER-positive BC [144]. The combination of the two agents results in a prolonged quiescent-like state, instead of exacerbation of the senescence-like phenotype. While palbociclib-resistant cells reactivate the CDK-RB-E2F pathway, inhibition of three pathways (i.e., CDK, mTOR, ER) was crucial for preventing resistance in MCF-7 xenograft models. Comparable effects were described in HNSCC models treated with a combination of abemaciclib and mTORi [25]. However, the effect on the immune system remains unclear. Given their broad application in preventing transplant rejection, global immune suppression instead of activation can be expected and constitutes a significant contraindication in cancer treatment.

Finally, a preexisting rare PIK3CA^E545K^ subpopulation was identified in an *NRAS*-melanoma patient upon combined CDK4i and MEKi treatment [145]. In a longitudinal analysis, this *PIK3CA*^E545K^-mutated clone was responsible for the relapse after an initial response. Hence, such subclones can rapidly expand to become the dominant resistant clone. To overcome resistance, an *in vitro* model of *NRAS*-mutant cutaneous melanoma cells was applied. In this model, specific S6K1 inhibition with the highly specific S6K1i PF-4708671 resensitized PIK3CA^E545K^ cells to the CDK4i and MEKi combination [145]. However, the S6K1 inhibition effects have to be confirmed in a larger cohort.

TP53 mutation or inactivation (by MDM2 interaction) was lately identified as a potential biomarker to predict abemaciclib resistance in BC patients (~ 25%). The same can be expected in other cancers, such as HNSCC harboring *TP53* alterations in up to 80% of patients. A strategy to reactivate p53 may be CDK9i [146]. In this context, knockdown of a negative p53 regulator—the inhibitor of apoptosis-stimulating protein of p53 (iASPP)—restored p53 function. Oncoprotein iASPP binds the C-terminus of *TP53* and inhibits its transcriptional activity. Still, iASPP does not bind mutant *TP53* and restricts this treatment to TP53 wild-type.

*TP53* wild-type is also required for palbociclib and abemaciclib to convert radioresistance [147]. Both inhibitors suppressed DNA damage repair, determined by increased γH2AX level in various TP53 wild-type models [147, 148]. Abemaciclib additionally affected mTOR signaling, HIF-1 expression, and irradiation-induced vasculogenesis *via* SDF-1 inhibition in xenograft models. Another interesting finding was the identification of inhibited DNA damage response gene *ataxia telangiectasia mutated* by palbociclib. Hence, radiosensitization is independent of CDK4/6 inhibition but induces “off-target effects” that may affect other molecular mechanisms. Remarkably, ribociclib is not radiosensitizing [148]. In GBM, *PTEN* wild-type may act as a predictor for the response, associated with suppressed Akt/ERK signaling [149].

A more holistic view of both tumor cells and the TME is crucial for treatment refining. Single-cell sequencing may guide the way to examine relapsed-resistant tumors. Based on this technique, acquired resistance to CDK4/6i and trastuzumab quickly emerged after the initial response in a transgenic mouse model [138]. Of note, a distinct immunosuppressive immature myeloid cell population, resembling MDSC characteristics, was identified. Targeting or modulating those cells to subvert the immunosuppressive TME into an inflamed environment even restored the vulnerability of highly aggressive BC to immune-checkpoint blockade and deserves further analysis.

## Conclusions and future perspectives

Predictive biomarkers of response and resistance are partially identified, yet they do not meet the clinical requirements for HNSCC and GBM. For HNSCC, HPV negativity constitutes the so far only established biomarker. Still, individual responses described in HPV-positive models warrant further investigation. Preclinical and clinical data for GBM are encouraging in some cases, but too limited to be transferred into clinical practice. To succeed, combinations with other agents are warranted. Therefore, sensitizing tumor cells to chemo- or radiotherapy and simultaneously stimulating the immune system with CDKi is promising to prevent or counteract resistance mechanisms. For the latter, a battery of targeted therapeutics is already available, and this number increases with the growing understanding of activated targets under the pressure of CDKi. Finally, treating patients with CDKi first-line—eventually in combination with immune-checkpoint inhibitors—will hopefully improve overall response.

## Electronic supplementary material

Table S1 (DOCX 88 kb)

Table S2 (DOCX 118 kb)

## Abbreviations

BBB: blood-brain barrier
BC: breast cancer
BCL: B cell lymphoma
CDK: cyclin-dependent kinase
CDKi/-’s: cyclin-dependent kinase inhibitor/-s
ctDNA: circulating tumor DNA
EGFR: epithelial growth factor receptor
ER: estrogen receptor
GBM: glioblastoma multiforme
HNSCC: head and neck squamous cell carcinoma
HPV: human papillomavirus
HR: homologous recombination
iASPP: inhibitor of apoptosis-stimulating protein of p53
LNP: lipid nanoparticles
MDSC: myeloid-derived suppressor cells
p-RB1: RB1 phosphorylation
RB1: retinoblastoma 1
STable: supplementary table
SCP3: synaptonemal complex protein 3
SUMO-1: small ubiquitin-like modifier-1
TAMC: tumor-associated myeloid cells
TME: tumor microenvironment
TMZ: temozolomide
Treg: regulatory T cell
