# Supplementary material for: Cyclin-dependent kinase inhibitors in head and neck cancer and glioblastoma—backbone or add-on in immune-oncology?
Source: Cancer Metastasis Rev. 2020 Nov 8;40(1):153–71. doi: 10.1007/s10555-020-09940-4 (PMC7897202; doi:10.1007/s10555-020-09940-4)
Supplement: Supplementary file 1 — (DOCX 88 kb) [file 10555_2020_9940_MOESM1_ESM.docx]

**Supplementary information for “Cyclin-dependent kinase inhibitors in head and neck cancer and glioblastoma - backbone or add-on in immune-oncology?” by Riess et al.**

Supplementary Table 1A. Preclinical approaches to target HNSCC.

| Study setting  Reference | Tumor entity/entities | Intervention | Treatment schedule | Methods | Outcome |
| --- | --- | --- | --- | --- | --- |
| *in vitro, in vivo*  [29] | HPV-negative HNSCC | palbociclib + Cisplatin (IC_50_) to generate resistant cells  🡪 BET-inhibitor JQ1 | palbociclib p.o. 70 mg/kg bw, daily  +/- JQ1, i.p. 70 mg/kg bw, daily | - Cisplatin-resistant cell line generation - immunoblot - cell cycle/proliferation/cell death analyses - DNA damage comet assay - Tumor xenografts | - palbociclib causes cell cycle arrest & decreased pRb in Cisplatin-sensitive cells - palbociclib increased cyclin E and CDK2 activity - Cisplatin-exposed cells exhibited diminished comet tails 1- and 4-h after radiation 🡪 higher levels of DNA repair - upregulation of c-Myc after Cisplatin exposure - prior Cisplatin exposure induces resistance to palbociclib *in vivo*, but not *in vitro* - Cisplatin-resistant samples show CyclinA/D alterations |
| *in vitro, in vivo*  [85] | nasopharyngeal carcinoma (NPC), EBV-positive or negative | AT7519, Cisplatin | *in vitro:* AT7519: 200 nM  Cisplatin: 20 μM  *in vivo:*  7.5 mg/kg or 15 mg/kg of AT7519 daily, 0.5 mg/kg Cisplatin every alternative day or the combination i.p. | - NPC cell lines and generation of chemo-resistant cell line - viability assay (Annexin V/7-AAD) - BrdU proliferation assay - combination analysis - anchorage-independent growth in soft agar - immunoblot - tritiated uridine assay - tumor xenografts | - dose-dependent inhibition of proliferation & colony formation  - induction of apoptosis  - synergism with Cisplatin and sensitization of chemo-resistant NPC cells  - inhibited transcription in both chemo-sensitive and chemo-resistant NPC cells  - overcomes NPC chemo-resistance *in vivo* |
| *in vitro, in vivo*  [57] | HNSCC (Cal27, FaDu, HN6) | THZ1, JQ1 | *in vitro:*  THZ1: 50 nM; JQ1: 2 μM  *in vivo:*  JQ1: 50 mg/kg bw, 5 days/week  THZ1: concomitantly, 10 mg/kg bw | - drug combination screening *in vitro* - viability assay (CCK-8) - colony formation assay - cell apoptosis (AnnexinV/PI) - senescence β-galactosidase - immunoblot - 4-nitroquinoline 1-oxide-induced HNSCC mouse model - HNSCC xenograft - immunohistochemistry - RNA-sequencing - prognostic score development and validation | - synergism of THZ1 and JQ1 likely because of BRD4/CDK7 inhibition - combination promotes cell apoptosis, senescence and modulates transcription of genes/multiple cancer-related pathways in vitro - combination impairs tumor growth and progression in vivo - development and validation of a prognostic score |
| *in vitro, in vivo*  [87] | human NPC cell lines (HONE-1, CNE-2, C666-1, and HK-1); EBV positive and negative | roniciclib, Cisplatin | *in vitro*: IC_50_ values determined  Cisplatin: 50 mg/mL  roniciclib: 10 mmol/L  -> *in vivo*: 40:60 mixture | - MTS cell proliferation/cell cycle/apoptosis assay - immunoblot - *in vivo* studies | - NPC cells = sensitive to roniciclib at clinically relevant doses - roniciclib inhibits multiple CDKs and their substrates and augments cytotoxicity of cisplatin in NPC cells but not in immortalized cells - roniciclib synergizes with Cisplatin in NPC xenografts |
| *in vitro*  [61] | HNSCC cell lines (SAS, FaDu, HSC4, Cal33, UTSCC5) | ZK304709  (pan-CDK inhibitor)  ± radiotherapy (RT) | ZK304709: 50 nM | - generation of CDK9-EGFP transfectants - colony formation assay - RT (8 Gy) - Small interfering (si)RNA-mediated CDK9 knockdown - immunofluorescence (γH2AX/p53BP1) - DAPI staining (apoptosis analysis) - immunoblot - cell cycle assay | - CDK9 overexpression enhances the clonogenic survival - CDK9 knockdown radiosensitizes HNSCC cells, impacts cell cycle protein expression, and modulates cell cycling in SAS and FaDu cells - CDK9 depletion fails to modulate DNA repair proteins - pharmacological inhibition of CDK9 mediates cytotoxicity but not radiosensitization |
| *in vitro, in vivo*  [152] | HNSCC cell lines (FaDu, Detroit-562 and SCC-25) | riviciclib  (P276-00) | *in vitro*: IC_50_ values determined  *in vivo*: P276-00, 50 mg/kg i.p., for 18 days | - xenograft model - cell viability assay (CCK-8) - cell cycle analysis - mRNA expression assay - immunoblot - Cell based automated fluorescence imaging - ELISA - immunohistochemistry of tumor xenografts | effects described on FaDu cells/xenografts:   - *In vitro* activity: G1 arrest & apoptosis induction via p53 phosphorylation - inhibited growth signaling proteins and cytokines - *in vivo*: tumor delay |
| *in vitro*  [10] | HNSCC cell lines (UM-SCC-22A, UM-SCC-38, VU-SCC-096,VU-SCC-120, FaDu), primary oral fibroblasts and keratinocytes | KU-60019, Wortmannin, ETP-46464, VE-821, MK-8776 (SCH 900776), PF-477736, LY2603618/  rabusertib, palbociclib), LY2606368/  prexasertib, adavosertib | different concentrations of indicated inhibitors | - lethality scores, siRNA transfections, viability assays - quantitative reverse transcription PCR (RT-qPCR) - expression microarray - whole-genome sequencing for 9p21.3 - dose-response curves with small molecule inhibitors - flow cytometry (DNA content) - time-lapse microscopy - immunoblot - apoptosis/necrosis/viability assay (ApoTox-Glo™ Triplex Assay) - metaphase analysis | specific Chk1 inhibition:   - impacts viability of HNSCC cells   - induces S-phase arrest & bimodal cell killing (time-lapse microscopy)  - activates caspase 3/7, or induces chromosomal breakage   - CDK1 levels = indicative for response |
| *in vivo*  [153] | NP69 (T antigen immortalized nasopharyngeal epithelial (NP) cells), C666–1 (NPC cell harboring EBV) and HK-1 (NPC cell without EBV) | Gemcitabine, decitabine, palbociclib | mean IC_50_ values | - cell growth assay - PDX models - drug sensitivity tests - genomic DNA extraction - whole exome sequencing - ACTOnco comprehensive Cancer panel sequencing (ACTgenomics) - copy number variation (CNV) analysis - RNAseq | - confirmation of *CCND1* overexpression via WES and CNV - NPC-PDXs drug screening - transcriptomic analysis of NPC PDX-B with various drug treatments - correlation of CNVs in *CCND1* and *CDKN2A* with EBV DNA load in NPC patient plasma - elevated *CCND1* expression = poor prognostic marker and potential treatment of PAL in NPC tumors |
| *in vitro*  [[122]](https://www.ncbi.nlm.nih.gov/pubmed/32117235) | HNSCC cell lines: FADU, Detroit-562, Cal-33, PE/CA/PJ-15, UT-SCC-14, UT-SCC-15 and patient-derived GBM cell lines | 5-FU, Cisplatin, Gemcitabine, Cetuximab, dinaciclib | 5-FU: 2.5 μM, Cisplatin: 0.2 μM, Gemcitabine: 0.0002 μM, Cetuximab: 0.34 μM; dinaciclib: 10 or 100 nM | - IFNγ stimulation - apoptosis/necrosis assay - hemolysis assay - IDO1 immunofluorescence - IDO1 immunohistochemistry on patients’ tumor samples - quantification of Tryptophan, Kynurenine, Kynurenic Acid in cell culture supernatant by liquid chromatography Tandem Mass Spectrometry System - RNA Isolation, cDNA synthesis, RT-qPCR | - basal IDO1 and related genes in GBM and HNSCC cell lines - gene expression and protein changes upon IFNγ stimulation - dinaciclib interferes with the Kynurenine pathway and blocks IFNγ-induced IDO1 expression in GBM and HNSCC cells - treatment induced influence on KP-related metabolites |
| *in vitro*  [19] | UT-SCC cell lines, UM-SCC-47 and 93- VU-147T, OE21 cells and other cell line entities | palbociclib  radiotherapy (RT) | palbociclib: 0.5 μM  RT: 2 Gy | - Immunoblot - RT-qPCR - cell cycle phase distribution/mitotic index - immunofluorescence - immunohistochemistry - metaphase spreads - homologous recombination assay - colony survival assays | - radiosensitization under hypoxic conditions/persistent DNA damage after radiation - effects on cell cycle are HPV-dependent - deficiency in homologous recombination - combination results in mitotic defects |
| *in vitro*  [28] | human HNSCC cell lines SCC4 and SCC25 and other cell line entity | ribociclib (LEE011)  radiotherapy | LEE011: 5-100 nM  RT: 4 Gy | - WST-1 cell proliferation/cell survival clonogenic assay - cell cycle profiling - immunoblot | - cell cycle arrest - inhibited pRB - no cytotoxicity in oral cancer cells - RT with LEE011 blocked cell cycle progression and pRB |
| *in vitro, in vivo*  [69] | human HNSCC cell lines | flavopiridol | *in vitro*: flavopiridol: 1-1000 nM  *in vivo:* 5 mg/kg bw per day, i.p. for 5 days | - thymidine incorporation - cell cycle analysis - DNA fragmentation assay - TUNEL analysis and DAPI staining - Tumor xenografts - immunohistochemistry | - antiproliferative effect/impaired cell cycle - inhibition of cdc2/cdk2 activity and cyclin D1 expression - apoptosis induction - 60-70% reduction in tumor size *in vivo,* enhanced apoptosis and altered cell cycle gene expression |

Supplementary Table 1B. Overview on running or recently completed clinical studies on HNSCC.

| Phase/Study design | Tumor entity/entities | Intervention | Treatment schedule and outcome measures | Status | *Clinical trial.gov identifier* |
| --- | --- | --- | --- | --- | --- |
| 2  Interventional, non-randomized | Metastatic or Recurrent HNSCC  Cohort 1: Not Previously Treated  Cohort 2: Treated Previously with immunotherapy | abemaciclib + pembrolizumab | - Cohort 1: - abemaciclib 150mg p.o. twice daily pembrolizumab 200mg IV every 3 weeks - Cohort 2: - abemaciclib 150mg p.o. twice daily pembrolizumab 200mg IV every 3 weeks - determine RR, AE grade 3 or higher of tumor lesions during different time frames - assess PFS/TTR/DR during different time frames | Active, not recruiting | NCT03938337 |
| Early Phase 1  Interventional | HPV negative HNSCC | ribociclib | - ribociclib 600mg, days 1-14, with surgical resection on day 14 - labs (coagulation parameters and others) and ECG = checked by days 9-12 - outcome measures: - decreased pRB1 levels - change in pre- to post-treatment Ki67/ERK1/2/p-ERK1/2/p16 in tissue samples | Terminated | NCT03179956 |
| 2  Interventional | 32 participants, recurrent or metastatic HNSCC who failed to Platinum-based therapy | abemaciclib | - abemaciclib 200mg p.o. twice daily - determine RR, PFS, OS DR and toxicity | Recruiting | NCT03356587 |
| 1, 2  Interventional  dose-escalation | 33 participants, HNSCC locally advanced | palbociclib + Cetuximab and RT (IMRT = Intensity Modulated Radiation Therapy) | - IMRT = administered 5 days on/2 days off (Σ: 70 Gy) for 33-35 fractions - Cetuximab 400 mg/m^2^ IV, 7 days before (day -7) starting radiation 🡪 then 250 mg/m^2^ IV weekly (7 weeks) - palbociclib = p.o., daily 3 week-on and 1-week off during IMRT (day 1-21 and day 29-49) on 3 dose levels + MTD - palbociclib:  1. dose Level 1: 100 mg p.o. every other day 3 week-on and 1-week off during IMRT 2. dose Level 1: 75 mg p.o. daily 3 week-on and 1-week off during IMRT 3. dose Level 2: 100 mg p.o. daily 3 week-on and 1-week off during IMRT 4. dose Level 3: 125 mg p.o. daily 3 week-on and 1-week off during IMRT  - Cetuximab: - all dose levels: 400 mg/m^2^ IV at 7 days before (day -7) starting radiation 🡪 then 250 mg/m^2^ IV weekly (7 weeks) - IMRT: - 5 days on/2 days off, (Σ: 70 Gy) for 33-35 fractions - determine DLTs, MTD and RP2D - determine efficacy, antitumor activity and safety profile of the combination (ORR) | Recruiting | NCT03024489 |
| 1  Interventional  dose-escalation | 96 participants, HNSCC and other solid tumors | palbociclib + gedatolisib | - palbociclib: - p.o. once daily, 3 weeks out of every 4 in each cycle - initial dose for part 1 = 100 mg daily - dosing adjusted until MTD and RP2D are established - gedatolisib: - i.v. once weekly on the first day for each of the four weeks during the 4-week cycle - initial dose for part 1 = 110 mg - dosing adjusted until MTD and the RP2D are established - assess MTD, RP2D, PFS and OS - target engagement of palbociclib and gedatolisib in paired tumor biopsies - determine different PK parameter | Recruiting | NCT03065062 |
| 1  Interventional  dose-escalation  single arm  (RISE-HN) | Recurrent and/or metastatic HNSCC | ribociclib + spartalizumab  (anti-PD1 antibody) | - ribociclib: p.o 400mg, 600mg, or 200mg daily, d1-d21, 28 days a cycle - spartalizumab: iv 400mg on d1, 28 days a cycle - assess AE, ORR, PFS, OS, DR, and ORR | Recruiting | NCT04213404 |
| 1  Interventional  Non-Randomized  dose-escalation | 126 participants, HNSCC and other advanced solid tumors | ribociclib + TNO155 (SPH2 Inhibitor) or spartalizumab + TNO155 | - TNO155: capsule + spartalizumab: concentrate for solution for infusion - TNO155: capsule + ribociclib: capsule and tablet - determine DLT, (serious) and AE - assess dose interruptions, reductions and dose intensity, by treatment - determine different PK parameter - assess efficiency (OS, PFS and DR) by treatment or combination | Recruiting | NCT04000529 |
| 2  Interventional  Non-Randomized (TRIUMPH) | 259 participants, HNSCC | Arm1: BYL719  Arm2: poziotinib (pan-HER-inhibitor)  Arm3: nintedanib (TKI)  Arm4: abemaciclib  Arm5:durvalumab, tremelimumab | - BYL719: p.o 100 mg once daily - poziotinib: p.o 12 mg once daily - nintedanib: p.o, initial dose 200 mg twice per day - abemaciclib: p.o, 200mg twice daily - durvalumab: 1.5g Q4W + Tremelimumab: 75mg Q4W up to 4 cycle then durvalumab 750mg Q2W, till PD or unacceptable toxicity - determine: DCR, RR, ORR, PFS, OS, TTP, quality of life assessment, duration of response, toxicity and biomarker analysis | Recruiting | NCT03292250 |
| 2  Interventional  Non-Randomized (The MATCH Screening Trial) | 6452 participants, HNSCC and solid tumors or lymphomas that have progressed following at least one line of standard treatment or for which no agreed upon treatment approach exists | a variety of targeted agents | Patients are assigned to 1 of 37 treatment subprotocols based on molecularly-defined subgroup  🡪 please see *clinicaltrial.gov identifier* for details | Recruiting | NCT02465060 |
| 2  Interventional  Randomized | 124 participants, HPV-negative, cetuximab-naive patients with recurrent/metastatic HNSCC | Arm1: palbociclib + Cetuximab  Arm2: placebo + Cetuximab | Arm 1:   - palbociclib = p.o. 75 mg, 100 mg, or 125 mg - day 1 to day 21 followed by 7 days off treatment in a 28-day cycle - cetuximab = iv 400 mg/m^2^ initial dose followed by 250 mg/m^2^   Arm2:   - cetuximab = iv infusion 400 mg/m2 initial dose followed by 250 mg/m2 - Placebo for palbociclib will be indistinguishable from the palbociclib capsules, same administration as palbociclib capsules - determine OS, PFS, CR, DR, AE, clinical benefit response and quality of life assessment | Active, not recruiting | NCT02499120 |
| 1  Interventional  dose-escalation | 24 participants, recurrent or metastatic HNSCC | palbociclib, avelumab, Cetuximab | - palbociclib: p.o. 125 mg, 100 mg, and 75 mg, depending on dosage daily, days 1-21 of 28 day cycle - avelumab: iv on days 1 and 15 of 28 day cycle - cetuximab iv 400 mg/m2 x 1, then weekly - assess MTD, ORR, PFS, OS and AE | Recruiting | NCT03498378 |
| 2  Interventional  Non-Randomized  (Los Tres Paso Trial) | 29 participants, HPV-unrelated HNSCC | palbociclib, followed by chemoradiation (either Cisplatin + IMRT or Cetuximab + IMRT depending on patient characteristics), followed by adjuvant single-agent palbociclib | Cohort 1:  1: palbociclib, 2: Cisplatin & IMRT, 3: palbociclib   - Step 1: Neoadjuvant palbociclib monotherapy p.o 125 mg/day, Days 1-21 of a 28-day cycle for two cycles - Step 2: Cisplatin iv 100 mg/m^2^ given on Days 1 and 22 with accelerated IMRT 70 Gy over 6 weeks - Step 3: Adjuvant palbociclib p.o 125 mg/day, days 1-21 of each 28-day cycle for six cycles; 16 to 22 weeks following completion of Cisplatin & IMRT   Cohort 2:  1: palbociclib, 2: cetuximab & IMRT, 3: palbociclib   - Step 1: Neoadjuvant palbociclib monotherapy p.o 125 mg/day, Days 1-21 of a 28-day cycle for two cycles - Step 2: Cetuximab given one week before RT and then weekly with accelerated IMRT 70 Gy over 6 weeks - Step 3: Adjuvant palbociclib p.o 125 mg/day, days 1-21 of each 28-day cycle for six cycles; 16 to 22 weeks following completion of cetuximab & IMRT - determine TTR of newly diagnosed p16^INK4a^ negative, HPV-unrelated HNSCC to neoadjuvant palbociclib monotherapy - assess PFS and OS | Recruiting | NCT03389477 |
| 2  Interventional | 21 participants, unresectable recurrent or metastatic HNSCC | palbociclib + Carboplatin | - palbociclib and Carboplatin for up to 6 cycles: - palbociclib p.o., dose= 125 mg daily, days=1-14, cycle length: 21 days - Carboplatin i.v., dose= AUC 5, day= 1, cycle length: 21 days - maintenance palbociclib after 6 cycles palbociclib p.o. 125 mg daily, days 1-21, cycle length: 28 days - determine DCR, PFS and OS | Completed | NCT03194373 |
| 1,2  Interventional  Non-Randomized | 96 participants, incurable HNSCC | palbociclib + Cetuximab | Dosel level 1 and 2:   - palbociclib days 1 through 21 of each 28 day cycle - Cetuximab i.v. (weekly schedule), first dose 400 mg/m^2^, remaining weekly dose 250 mg/m^2^,weekly cetuximab (250 mg/m^2^) for duration of participation on study   Phase II Arm 1: Platinum-resistant HPV-unrelated HNSCC  Phase II Arm 2: Cetuximab-resistant HPV-unrelated HNSCC   - Same treatment regime in dose level 1 and 2 - determine MTD, ORR in different groups - assess AE, PFS, OS and duration of stable disease | Active, not recruiting | NCT02101034 |
| 2  Interventional  Non-Randomized  (UPSTREAM) | 340 participants, recurrent or metastatic HNSCC progressing after first-line Platinum-based chemotherapy | palbociclib, afatinib, Standard of care (SOC); IPH2201 (monalizumab); durvalumab; niraparib; BAY1163877 (rogaratinib) | - palbociclib p.o. 125 mg, once daily, 1 cycle is 28 days (21 days on treatment, then 7 days off - afatinib p.o. 40 mg, once daily, 1 cycle is 28 days - niraparib p.o., 300 mg, once daily, 1 cycle is 28 days - rogaratinib p.o., 600 mg, twice daily, 1 cycle is 28 days - monalizumab i.v., 10mg/kg, once every 14 days, 1 cycle is 14 days or - monalizumab i.v., 750mg, once every 28 days, 1 cycle is 28 days - durvalumab i.v., 1500mg, once every 28 days, 1 cycle is 28 days - 8 different patient cohorts, please see *clinicaltrial.gov identifier* for details - determine PFS rate and ORR at week 16 - assess PFS and ORR, DR, OS and AE - percentage of each patient cohort according biomarker testing - patients with an evaluable fresh tumor biopsy | Recruiting | NCT03088059 |
| 1  Interventional  Non-Randomized | 90 participants, Head and neck cancer and solid tumors | palbociclib + Cisplatin  palbociclib + Carboplatin | Arm1:   - Cisplatin i.v. on day 1 and palbociclib p.o. once a day on days 2-22 🡪 treatment repeats every 28 days for up to 6 courses   Arm2:   - Carboplatin i.v. on day 1 and palbociclib p.o. once a day on days 2-22 🡪 treatment repeats every 28 days for up to 6 courses - determine AE, DLTs, RP3D, ORR, and PK characteristics | Recruiting | NCT02897375 |
| 1,2  Interventional  Non-Randomized | 32 participants, recurrent/metastatic HNSCC that progressed or recurred within six months after Platinum-based chemotherapy | abemaciclib + nivolumab | Experimental: Phase I:   - abemaciclib p.o. twice per day on days 1 through 28 of every 4-week cycle   Experimental: Phase II:   - abemaciclib monotherapy = recommended phase II dose on day -7 through day -1 prior to starting cycle 1 with abemaciclib + nivolumab - proceed directly from day -1 to cycle 1 day 1 of abemaciclib + nivolumab - treated with abemaciclib at the RP2D (Days 1 through 28) + nivolumab (480 mg, day 1) of each 4-week cycle - determine RP2D and OS - assess best tumor response, DR, PFS and AE and changes in peripheral blood lymphocyte subsets | Recruiting | NCT03655444 |
| 2  Interventional  (ABORL) | 25 participants, locally advanced/metastatic HNSCC after failure of Platinum and Cetuximab or anti-EGFR-based therapy and harboring an homozygous deletion of CDKN2A, and/or an amplification of CCND1 and/or of CDK6 | abemaciclib | - abemaciclib p.o, 400mg/day with 2 doses of 200mg 12-hour apart for each 28-day cycle, Σ 56 doses of study drug - determine CR, PR and SD - assess ORR, DR, best response rate, time to treatment failure, PFS and OS | Recruiting | NCT03356223 |
| 2  Interventional  Randomized | 20 participants, operable HPV-negative HNSCC | abemaciclib, abemaciclib + nivolumab | Arm1:   - abemaciclib p.o, 150 mg, twice daily on days 1-21 (+7 days)   Arm2:   - abemaciclib p.o, 150 mg, twice daily on days 1-21 (+7 days) + nivolumab i.v., 240 mg on days 1 and 15 | Not yet recruiting | NCT04169074 |
| 1  Interventional  dose escalation  (CETLEE011) | 21 participants, recurrent or metastatic HNSCC | ribociclib (LEE011) + Cetuximab | - LEE011 p.o 400 mg / 600 mg daily + Cetuximab to determine MTD and toxicity profile | Terminated | NCT02429089 |
| 1/2 Interventional, multicenter (SPARK) | 23 participants; recurrent and/or locally advanced HNSCC | P276-00 + radiation (external beam radiotherapy (EBRT)) | P276-00 iv:   - dose level 1: 100 mg/m^2^/day dose - dose level 2: 140 mg/m^2^/day - dose level 3: 185 mg/m^2^/day   🡪 from day 1 to day 5 every 21 days for 2 cycle; from days 1 to 5 per 21 day cycle for two cycles  External beam radiotherapy (EBRT):   - standard conventional fractionation i.e. 2 Gy per day for 5 days a week; Σ 60 Gy over 6 weeks followed by up to 10 additional Gy if required - determine the MTD, DLTs and PK of P276-00 - determine safety, tolerability and efficacy of combination regime   - analysis of biomarkers associated with use of P276-00 and radiation | Completed | NCT00899054 |
| Interventional, multicenter phase 2 study (MONARCH) | 86 participants, recurrent and/or Locally Advanced HNSCC | riviciclib (P276-00) | - P276-00 iv, 144 mg/m^2^/day from day 1 to day 5 and from day 8 to day 12 in each 21 day cycle - response rate and evaluation (measurements at baseline and at the end of 2 cycles) - duration of response, PFS and OS at one year (measurements at the end of 2 cycles; follow up every 4 weeks) | Completed | NCT00824343 |
| 2 | 12-37 patients, recurrent or metastatic HNSCC | flavopiridol, aspirin, alvocidib, clopidogrel bisulfate | - flavopiridol i.v. on days 1-5, repeat every 3 weeks - aspirin and clopidogrel bisulfate p.o day 0 and continuing throughout the study - determine RR and DR, efficiency, PFS and OS - determine anti-platelet agents, aspirin and clopidogrel bisulfate, on the pharmacology of flavopiridol - determine prophylactic anticoagulation with anti-platelet agents, aspirin and clopidogrel bisulfate, on the incidence of flavopiridol-related thrombosis | Completed | NCT00020189 |

p.o: per oral, iv: intravenous, IMRT: Intensity Modulated Radiation Therapy, MTD: Maximum Tolerated Dose, AE: adverse events, RP2D: Recommended Phase 2 Dose, PFS: progression free survival, OS: overall survival, PK: pharmacokinetics, iv: intravenous, DCR: Disease control rate, RR: Response rate, ORR: Overall response rate, TTR: time to tumor response, TTP: time to progression, DR: duration of response, DLT: dose limiting toxicity

Supplementary Table 1C: Overview on completed clinical studies

| Phase/  Study design/  Reference | Tumor entity/entities | Intervention | Treatment schedule | Treatment-related toxicities | Outcome |
| --- | --- | --- | --- | --- | --- |
| 1  [30] | recurrent/metastatic HNSCC  9 patients (five p16^INK4a^ negative; four positive) enrolled across dose levels 1 (n=3) and 2 (n=6)  6/9 patients had cetuximab-resistant and 4/9 had Platinum-resistant disease | palbociclib + Cetuximab | - determine DLT and MTD of palbociclib with standard dose weekly Cetuximab - palbociclib = p.o, days 1-21 every 28 days: - dose level 1 100 mg/d - dose level 2 125 mg/d; approved monotherapy dose | myelosuppression | - DC: 89%, including PR: 22% and SD: 67% - PRs occurred p16^INK4a^ negative HNSCC - 56% = decreases in tumor target lesions - Cetuximab-resistant HNSCC: PR in 1 and DC in 5, TTP = 112 days - Platinum-resistant HNSCC: PR in 1, DC in 3, TTP = 112 days - palbociclib 125 mg/day on days 1-21 every 28 days with Cetuximab was safe - Tumor responses = observed in Cetuximab- or Platinum-resistant disease |
| 2  [31] | HPV-unrelated HNSCC  platinum-resistant (group 1)  cetuximab-resistant (group 2) | palbociclib + Cetuximab | - palbociclib p.o, 125 mg/day, on days 1–21 + Cetuximab i.v., 400 mg/m^2^ on cycle one, day 1, then 250 mg/m^2^ once per week in 28-day cycles | neutropenia (34%)  no treatment-related death | - Platinum-resistant or Cetuximab-resistant HPV-unrelated HNSCC = palbociclib + Cetuximab = promising activity outcomes: - group 1: OR 39% - group 2: OR 19% |
| 1  classical 3 + 3 dose escalation  **[154]** | 21 patients, R/M HPV-negative HNSCC | ribociclib, Cetuximab | - ribociclib p.o, one dose level: 400 mg/day, second dose level: 600 mg/day, on days 1–21 of each 28-day cycle - Cetuximab iv, 400 mg/m^2^ on cycle 1 day 1, followed by 250 mg/m2 weekly | Grade 1-2 AEs: diarrhea (52%), rash (52%), fatigue (43%), nausea (33%), and mucositis (28%) grade 3–4 AE: neutropenia (20%) | - 10 patients enrolled in the escalation trial - no DLTs observed at dose level 1 - dose level 2: 1/6 patients experienced DLT (grade 4 thrombocytopenia) - PFS = 3.5 months and OS = 8.3 months; PR 10.5 % and SD 58% - recommended dose: 600 mg daily on a 3-weeks on and 1-week off schedule in combination with cetuximab |
| 1  open-label, nonrandomized, dose-escalation study  [155] | 48 patients, 1x HNSCC and other advanced malignancies | flavopiridol, Oxaliplatin and 5-FU/Leucovorin | - flavopiridol i.v., initially 40 mg/m^2^ + Oxaliplatin i.v., initially 60 mg/m^2^ and leucovorin (400 mg/m^2^) followed by 5-FU bolus (400 mg/m^2^) and continuous 5-FU starting dose 1,800 mg/m^2^, regimen administered every 2 weeks - Dose escalation with flavopiridol pursued in 10 mg/m^2^ intervals up to 80 mg/m^2^ | most common: grade 3 toxicities  grade 4 toxicities: hematologic, non-hematologic toxicities ≤20% of the time: fatigue, diarrhea, nausea, vomiting, electrolyte abnormalities, sensory neuropathy, and febrile neutropenia | - MTD = 70 mg/m^2^ flavopiridol, 85 mg/m^2^ Oxaliplatin, and 1,800 mg/m^2^ 5-FU continuous infusion over 48 hours - 33% PR, 70 % decline in serum tumor markers, PK = inter-patient variability - tumor samples: p53 mutant (>30% positive cells) for responders and p53 wild-type for non-responders |
| 1  open-label, non-randomized, dose escalation study  [156] | 34 patients, 1x esophageal and other solid tumors | flavopiridol and vorinostat | - flavopiridol 60–80 mg/m^2^ - Vorinostat p.o intermittent 400 mg/day - One week before combination treatment, Vorinostat alone was given daily for 3d 🡪 Vorinostat was given on d1-3 and d8-10, and flavopiridol on d2 and d9, every 21-d - Modified due to neutropenia: Vorinostat on d1-3 and d15–17, and F on d2 and d16, every 28-d | Anemia grad 3,4; thrombosis grade 4; infection grade 3; leucocytes grade 3 and 4; lymphopenia grade 3,4; neutropenia grade 3 and 4; thrombocytopenia grade 3 and 4 | - 21 d schedule 🡪 MTD: V = 600 mg/d, F = 60 mg/m^2^ bolus - 28 d schedule 🡪 MTD: V = 800 mg/d, F = 30 mg/m^2^ (30 min) and 30 mg/m^2^ (4h) - V: C_max_ (800 mg) = 4.8 µM (± 2.8) - V C_max_ ≥2.5 µM = (86%) at MTD - F increased the C_max_ of V by 27% (95% CI 11%–43%) - F C_max_ of ≥2 µM (90%) - SD = 8 patients for 5.5 m |
| 1  [157] | 55 patients, 2x HNSCC and other entities with refractory neoplasms | flavopiridol | - flavopiridol i.v., doses from 12 to 78 mg/m^2^ daily for 5, 3 and 1 day every 3 weeks | neutropenia, fatigue and secretory diarrhea | - median C_max_ = 1.7 (1.3–4.2) (n=9), 3.2 (1.7–4.8) (n=8) and 3.9 μM (1.8–5.1) (n=6), respectively, at doses of 37.5 mg/m^2^/day×5 days, 50 mg/m^2^/day×3 days and 62.5 mg/m^2^/day×1 day - AUC = linear, total clearance was 13.8±4.9 l/h/m^2^ - elimination half-life = 5.2±4.9 h - volume of distribution at steady state = 64.9±43.4 l/m^2^ - MTD: 50 mg/m^2^/day - DLT : secretory diarrhea - 10% homozygous and 22% heterozygous patients for UGT1A1*28 |
| window design clinical study  [73] | 20 patients, locally advanced (WHO type III ) nasopharyngeal carcinoma | seliciclib | - seliciclib p.o. 800 mg or 400 mg twice daily on days 1 to 3 and 8 to 12. | 800 mg: 1 patient grade 3 liver toxicity, 1 patient grade 2 vomiting | - 7 of 14 = tumor reduction 🡪 associated with tumor apoptosis, necrosis, decreases in plasma EBV post-treatment, reduced protein level of Mcl-1, cyclin D1, pRB, transcriptional downregulation of genes related to cellular proliferation and survival, inhibition of cdk2/cyclin E, cdk7/cyclin H, and cdk9/cyclin T - PK: 800 mg 🡪 peak plasma concentration: 6.6 ± 1.5 μg/mL - mean Tmax = 1.3 ± 0.6 hours - mean AUC 0-last of 22.8 ± 9.6 μg × h/mL on day 1 - 400 mg 🡪 mean peak plasma level = 1.3 ± 0.7 μg/mL - AUC 0-last = 4.5 ± 2.7 μg × h/mL |

DLT: dose limiting toxicity, TTP: time to progression, MTD: Maximum tolerated dose, DC: Disease control, PR: partial response, RP2D: recommended phase II dose; SD: Stable disease, AE: adverse events, p.o.: per oral

**References**

150. Mishra, P. B., Lobo, A. S., Joshi, K. S., Rathos, M. J., Kumar, G. A., & Padigaru, M. (2013). Molecular mechanisms of anti-tumor properties of P276-00 in head and neck squamous cell carcinoma. *Journal of Translational Medicine, 11* (1). <https://doi.org/10.1186/1479-5876-11-42>.

151. Hsu, C.-L., Lui, K.-W., Chi, L.-M., Kuo, Y.-C., Chao, Y.-K., Yeh, C.-N., et al. (2018). Integrated genomic analyses in PDX model reveal a cyclin-dependent kinase inhibitor Palbociclib as a novel candidate drug for nasopharyngeal carcinoma. *Journal of Experimental & Clinical Cancer Research : CR, 37* (1), 233.

<https://doi.org/10.1186/s13046-018-0873-5>.

152. Seront, E., Schmitz, S., Papier, M., Van Maanen, A., Henry, S., Lonchay, C., et al. (2019). Phase 1 study evaluating the association of the cyclin-dependent kinase 4/6 inhibitor ribociclib and cetuximab in recurrent/metastatic p16-negative squamous cell carcinoma of the head and neck. *Frontiers in Oncology, 9*(MAR). <https://doi.org/10.3389/fonc.2019.00155>.

153. Rathkopf, D., Dickson, M. A., Feldman, D. R., Carvajal, R. D., Shah, M. A., Wu, N., et al. (2009). Phase I study of flavopiridol with oxaliplatin and fluorouracil/leucovorin in advanced solid tumors. *Clinical Cancer Research, 15*(23), 7405–7411. <https://doi.org/10.1158/1078-0432.CCR-09-1502>.

154. Dickson, M. A., Rathkopf, D. E., Carvajal, R. D., Grant, S., Roberts, J. D., Reid, J. M., et al. (2011). A phase I pharmacokinetic study of pulse-dose vorinostat with flavopiridol in solid tumors. *Investigational New Drugs, 29*(5), 1004–1012. <https://doi.org/10.1007/s10637-010-9447-x>.

155. Zhai, S., Sausville, E. A., Senderowicz, A. M., Ando, Y., Headlee, D., Messmann, R. A., Arbuck, S., Murgo, A. J., Melillo, G., Fuse, E., & Figg, W. D. (2003). Clinical pharmacology and pharmacogenetics of flavopiridol 1-h i.v. infusion in patients with refractory neoplasms. *Anti-Cancer Drugs, 14*(2), 125–135.

<https://doi.org/10.1097/00001813-200302000-00006>.

156. Greenall, S. A., Lim, Y. C., Mitchell, C. B., Ensbey, K. S., Stringer, B. W., Wilding, A. L., et al. (2017). Cyclin-dependent kinase 7 is a therapeutic target in high-grade glioma. *Oncogenesis, 6*(5). <https://doi.org/10.1038/oncsis.2017.33>.

157. Long, F., He, Y., Fu, H., Li, Y., Bao, X., Wang, Q., et al. (2019). Preclinical characterization of SHR6390, a novel CDK 4/6 inhibitor, in vitro and in human tumor xenograft models. *Cancer Science, 110*(4), 1420–1430. <https://doi.org/10.1111/cas.13957>.

158. Dahl, N. A., Danis, E., Balakrishnan, I., Wang, D., Pierce, A., Walker, F. M., et al. (2020). Super elongation complex as a targetable dependency in diffuse midline glioma. *Cell Reports, 31*(1). <https://doi.org/10.1016/j.celrep.2020.03.049>.

159. Xie, Q., Wu, Q., Kim, L., Miller, T. E., Liau, B. B., Mack, S. C., et al. (2016). RBPJ maintains brain tumor-initiating cells through CDK9-mediated transcriptional elongation. *Journal of Clinical Investigation, 126* (7), 2757–2772. <https://doi.org/10.1172/JCI86114>.

160. Yin, T., Lallena, M. J., Kreklau, E. L., Fales, K. R., Carballares, S., Torrres, R., et al. (2014). A novel CDK9 inhibitor shows potent antitumor efficacy in preclinical hematologic tumor models. Molecular Cancer Therapeutics, 13(6), 1442–1456. https://doi.org/10.1158/1535-7163.MCT-13-0849.
