# Supplementary material for: Cyclin-dependent kinase inhibitors in head and neck cancer and glioblastoma—backbone or add-on in immune-oncology?
Source: Cancer Metastasis Rev. 2020 Nov 8;40(1):153–71. doi: 10.1007/s10555-020-09940-4 (PMC7897202; doi:10.1007/s10555-020-09940-4)
Supplement: Supplementary file 2 — (DOCX 118 kb) [file 10555_2020_9940_MOESM2_ESM.docx]

**Supplementary information for “Cyclin-dependent kinase inhibitors in head and neck cancer and glioblastoma - backbone or add-on in immune-oncology?” by Riess et al.**

Supplementary Table 2A. Preclinical approaches to target GBM.

| Study setting,  Reference | Tumor entity/entities | Intervention | Treatment schedule | Methods | Outcome |
| --- | --- | --- | --- | --- | --- |
| *in vitro, in vivo*  **[33]** | GBM cell lines: U87MG, U138MG, M059J, Hs683, H4, A172, LN18, LN229, CCF-STTG1, T98G, and DBTRG-05MG, 8MGBA, 42MGBA, DKMG, GAMG, GMS10, LN405, and SNB19, AM38, NMC-G1, and KG-1-C | üalbociclib in combination with radiotherapy (RT) | *in vitro*:  palbociclib: 1 µM  *in vivo:*  150 mg/kg bw/day 50 mM p.o., TMZ 10 mg/kg/day, radiotherapy (RT) = 2 Gy/day | - immunoblot - flow cytometry - senescence β-galactosidase - Rb knockdown - intracranial GBM xenograft - Brain tumor analysis (LC-MS/MS method) | Palbociclib:  - induces cell cycle arrest and senescence of Rb-proficient GBM cells   - alleviates the growth arrest in stable depleted of Rb expression - suppresses growth of GBM intracranial xenografts & enhances survival of mice in combination with radiation - effectively suppresses growth of recurrent GBM |
| *in vitro, in vivo*  **[34]** | GBM cell lines: GS2, GBM34 and GBM43 | palbociclib  in combination with RT | *in vitro*:  palbociclib: 0.001 - 10 µM 4 days  *in vivo:*  4 treatment groups:   - vehicle control = 50 mM sodium lactate, pH 4 - palbociclib p.o., daily 150 mg/kg bw for 14 consecutive days - RT: 1 Gy for 5 days - combination | - Immunoblot - cell proliferation/cell-cycle - clonogenic survival Assay - fluorescent immunocytochemistry - intracranial xenograft and bioluminescence Imaging - histology | - in vitro response to palbociclib - improved outcome after combination therapy *in vivo* |
| *in vitro, in vivo*  **[35]** | patient-derived cell lines: GBM-L1, HW1, RN1 and BAH1 cell lines | palbociclib | *in vitro*:  palbociclib (with and without RT): 4 μM for 48 h  or 0x, 0.5x, 1x or 1.5x their respective IC_50_ values for 72 h  *in vivo:*   - palbociclib p.o., 75 mg/kg/day for 5 days in a two week treatment cycle - RT: 4 Gy over 2 days - combination | - colony formation & cell proliferation assay - cell cycle analysis - apoptosis assay (Annexin V-) - immunoblot - intracranial mouse model | - palbociclib - is effective as a monotherapy - induces cell cycle arrest and apoptosis - synergizes with RT to prevent colony formation - combined with RT increases survival in an orthotopic GBM model |
| *In vitro*  **[37]** | Human fibroblasts (HF), GSC-ECLs = G02, G07, G08, and G09 (human GBM biopsies) | palbociclib, roscovitine, GSK690693 (pan-Akt inhibitor), rapamycin | palbociclib: 0.1 – 10µM  roscovitine: 1 – 20µM  GSK690693: 1µM  rapamycin: 100nM | - RT-PCR - immunoblot - cell viability assay (trypan blue) - flow cytometric analysis of BrdU Incorporation and cell cycle distribution - cell transfection & RNA interference - Immunostaining and fluorescence microscopy - senescence β-galactosidase - growth rate estimation | Effects on GSC-ECLs:  - CDK4/6 inhibition induces cell cycle arrest   - CDK6 mainly governs proliferation - sustained palbociclib treatment induces a senescent-like phenotype - palbociclib treatment does not eliminate replicative potential |
| *in vitro, in vivo*  **[38]** | proneural GBM | palbociclib and N,N-diethylaminobenzaldehyde (inhibitor of the mesenchymal driver ALDH1A3) | *in vitro*: palbociclib: 10 μM and 20 μM (or higher)  *in vivo*:  palbociclib, p.o. 150 mg/kg bw daily | - PCR/immunoblot  cell viability assay (trypan blue)flow cytometric analysis of BrdU Incorporation and Cell Cycle Distributioncell transfection and RNA Interferenceimmunostaining and fluorescence microscopySA-β-Gal Staininggrowth rate estimation  - intracranial xenografts of a proneural GSC line | - prolonged survival of mice  - combination: strong synergistic inhibitory effects |
| *in vitro, in vivo*  **[39]** | GBM cell lines: U87, patient‐derived GBM cells N33 | combination of palbociclib and AQB (HOTAIR-EZH2 inhibitor) | *in vivo*:  palbociclib, 100 mg/kg bw  AQB, 100 mg/kg bw or combination once every 2 days | protein mass spectrometryplasmid and siRNA transfectionimmunoprecipitation (ChIP) and ChIP‐qPCR analysisRNA sequencing and microarray data samplescell viability, colony formation, and transwell assay  - cell cycle distribution by flow cytometry  immunoblot and real‐time PCRH&E staining, immunohistochemistry, confocal imaging | - combination: more effective than either drug alone - synergistic blockade of the G1 phase - reduced migration and invasion by inhibiting Wnt/β‐catenin signaling |
| *in vitro, in vivo*  [41] | Diffuse intrinsic pontine glioma (DIPG) cell lines TT10630, TT10714, TT10728 and TT11201 | palbociclib, erlotinib | *in vitro*:  palbociclib: 6.25 nM - 25600 nM  *in vivo:*  - palbociclib, intragastrically 150 mg/kg/day for 21 days  - combination: palbociclib 150 mg/kg bw/day for 14 days, followed by 75 mg/kg bw palbociclib with 37.5 mg/kg bw erlotinib for 7 days | sphere formation assayorthotopic xenograft mouse modelsimmunoblot and RT-qPCR analysescell viability assaycell cycle analysis and flow cytometryapoptosis analysisHE staining and immunohistochemistryRNA-sequencing | - palbociclib effectively repressed DIPG growth in vitro - palbociclib has high efficiency of blocking tumor growth *in vivo* |
| *in vitro, in vivo*  **[18]** | DIPG cell lines: SJ-DIPGx7, Line 7 | ribociclib | *in vivo:*  ribociclib, p.o. 100 mg/kg | ribociclib protein binding in mouse plasmadevelopment of pharmacokinetic limited sampling modeling for ribociclib*in vitro* probe recovery studiescerebral microdialysis studiespharmacokinetic analysis of plasma and microdialysis studies | - mean unbound ribociclib plasma exposure (6812 ng/ml*hr) was similar to that observed clinically at recommended dosages in adults - median ribociclib ECF to plasma partition coefficient (K_p,uu_) in non-tumor and tumor-bearing mice: 0.10 and 0.07 |
| *in vitro, in vivo*  **[47]** | GBM and peripheral tumors with high incidence of brain metastases  cell lines: Madin-Darby canine kidney (MDCK) cells expressing human MDR1 or mouse bcrp1 | abemaciclib  palbociclib  temozolomide (TMZ) | *in vitro*:  IC_50_ value  *in vivo*:  test compound, i.v. by 2.2 μmol/kg bw | efflux transporter substrate and inhibition assaysdetermination of plasma protein and brain bindingLC-MS/MS analysis*in vivo* xenograft | - abemaciclib increased survival of intracranial U87MG tumor-bearing rats similar to TMZ - combination of abemaciclib and TMZ additive or greater than additive - abemaciclib crosses the blood-brain barrier |
| *in vitro, in vivo*  **[158]** | GBM  patient-derived neurosphere cell lines, melanoma cell lines A375, A2058, IPC-298, SK-MEL-28, CHL-1 and MeWo, BJ fibroblast line, HS5 bone marrow stromal cell line | THZ1 | *in vitro:*  1 – 500 nM  *In vivo*: 10 mg/kg i.p. | - proliferation assays - microarray analysis - high-resolution respirometry - cell cycle analysis - in vivo tumor intracranial xenografts | *in vitro*:   - perturbed transcriptome and disabled CDK activation - G2 cell cycle arrest/DNA damage - halted transcription of the nuclear-encoded mitochondrial ribosomal genes - reduced mitochondrial translation and oxidative respiration - inhibited EGFR and PDGFR-α expression - reduced signaling flux through the AKT, ERK1/2, STAT3 downstream pathways - disrupted nucleolar, cajal body and nuclear speckle formation, resulting in reduced cytosolic translation and malfunction of the spliceosome and thus leading to aberrant mRNA processing.   *in vivo*:  no increase in animal survival over 40 days 🡪 THZ1 not crossing blood–brain barrier |
| *in vitro, in vivo*  **[58]** | GBM cell lines U87 and U251, primary cell lines SHSMU_ GBM05 and SHSMU_GBM06 | collection of epigenetic modulating small molecules (including THZ1) | *in vitro*:  1 µM and 10 µM  *in vivo*:  THZ1 i.v. 10 mg/ kg twice daily | tumor xenograft modelscell cycle, proliferation and apoptosis assaysRNA sequencingchromatin immunoprecipitation sequencing (ChIP-seq) data processingsubcutaneous xenograft model | - disruption of global gene transcription |
| *in vitro*  **[59]** | GBM cells T98G | flavopiridol | 300 nM | affymetrix microarray hybridization, quantitation, hierarchical cluster analysis, visualizationprotein and RNA analyses | potent and rapid CDK9 activity inhibition |
| *in vitro, in vivo*  **[62]** | GBM cell lines U87, T98G, LN229, HS683, LN18, intestine epithelial cells (HIEC-6, CRL3266, GBM stem cells (GSC) including GSC923 and GSC827 cell lines | TG02 as single agent and in combination with TMZ | *in vitro*:  TG02 36 nmol/L and 58 nmol/L in these cell lines  *in vivo*:  TG02 p.o., 30 mg/kg twice a week  TMZ, p.o., 5 mg/kg daily for 5 days | cell viability assayimmunoblot analysiscolony formation assayraman imagingimmunofluorescence & electron microscopycell apoptosis detectionRNA sequencingintracranial GBM model, bioluminescence imagemitochondrial and glycolytic function assays | *in vitro*:   - inhibited cell proliferation, induced cell death - synergism with TMZ in GBM cells but not in astrocytes - cytotoxicity blocked by overexpression of pCDK9 - suppressed transcriptional progression of antiapoptotic proteins and induced apoptosis - mitochondrial dysfunction and glycolytic suppression, ATP depletion   *in vivo*:  - prolonged survival in the combination |
| *in vitro*  **[64]** | GBM cell lines U87-MG and U251-MG | PHA-767491 hydrochloride | 2.5 or 10 µM | cell proliferation/viability assaydetection of cell deathimmunoblottingreal-time PCRmigration/invasion assay | *in vitro*:   - CDC7 inhibition reduces GBM cell viability, suppresses cell proliferation, and triggers apoptosis in GBM cell lines - suppressed GBM cell migration and invasion |
| *in vitro*  **[65]** | GBM cell line LN-229, glioma-initiating cell lines (GIC), T-325, ZH-161, S-24, and ZH-305 | TG02  IFN-β | *in vitro*:  TG02 0.1 – 50nM  IFN-β 10 IU/ml | acute growth inhibition assayRT-qPCRimmunoblotflow cytometrycaspase activity assay | - pre-exposure to IFN-β sensitized to TG02 - induction of caspase-independent apoptosis by TG02 was converted by IFN-β pretreatment |
| *in vitro, in vivo*  **[77]** | GBM neurosphere line BT224-luc2 | seliciclib  CYC065 (CDK2/9 inhibitor)  drozitumab | *in vitro*:  seliciclib 30 μM  drozitumab 10 μg/mL  CYC065 5 μM  *in vivo*:  CYC065, p.o. 55 mg/kg for 2 weeks | cell viability assayflow cytometryPDX modelbioluminescent imagingimmunoblothistology and immunohistochemistry | - seliciclib + drozitumab: reduced viability and induction of apoptosis - growth inhibition in PDX models - but: lethal toxicity (45%) when dosing seliciclib to reach the brain |
| *in vitro*  **[78]** | GBM | roscovitine | 1–100 μM | determination of [^3^H]methyl thymidine incorporation into DNAdetermination of DNA synthesis rate and effect of roscovitine | - strong inhibitory effect of roscovitine on DNA synthesis rate |
| *in vitro*  **[79]** | GBM cell lines A172 and G28 | roscovitine | serial concentrations for 24-120 h | xCELLigence  - cell cycle analysis (flow cytometry)  RT-qPCRimmunoblot | dose-dependent effects:   - anti‑proliferative and pro‑apoptotic (A172 > G28) - increase of the G2/M and sub-G1 fractions - decrease of transcripts of p53, CDK7 and cyclins A and E and an increase of >4-fold of p21 |
| *in vitro*  **[80]** | GBM cell lines U87MG, T98G, A172, and U251MG | roscovitine | 20 μ M | cellular viability & clonogenic assayimmunoblot/cell cycle analysisimmune complex kinase assaycaspase-8 activity assaysemiquantitative RT–PCR | - inhibited Cdc2 activity, sensitization to TRAIL-induced apoptosis - downregulated the protein levels of survivin and XIAP |
| *in vitro, in vivo*  **[81]** | GBM cell lines U87, U373, LN18 and rat glioma cell line C6 | roscovitine TMZ | *in vitro*: roscovitine: 100nM, 250 nMTMZ: IC_50_ *in vivo:*  roscovitine 25 mg/Kg  TMZ 10 mg/Kg  Combination, drugs administered on alternate days in total 3 doses | MTT cytotoxicity assaylong-term survival assayimmunoblot/ immunofluorescencesemi-quantitative PCRanimal experiments (rats)histology and immunohistochemistry | - pre-treatment with roscovitine enhanced chemo-sensitivity and suppressed the GBM growth - reduced Cdk-5 activity, induction of autophagy and Caspase-3 mediated apoptosis - combination restricts GBM growth, reduces angiogenesis and eliminates reactive astrocytes |
| *in vitro, in vivo*  **[82]** | GBM cell lines LN229, U87, U373, SF767, human glioma GBM43 and GBM14 cells, human breast cancer cell lines BT549, MDAMB231, T47D, and BAX-WT, and BAX-KO MEFs | roscovitine  PIK-90  (PI3K inhibitor) | *in vitro*:  roscovitine: 25 μ M  PIK-90: 0.5 μM  *in vivo:*  PIK-90, i.p., 40 mg/kg daily  roscovitine 50 mg/kg  combination | - cell cycle analysis (flow cytometry)  immunoblotimmunohistochemistryxenografts | - roscovitine cooperates with PIK-90 - combination blocks survivin and drives cell death - combination well tolerated *in vivo* - synthetic-lethal manner 🡪 induce apoptosis in xenografts |
| *in vitro*  **[88]** | GBM cell lines | dinaciclib,  ABT-737, ribociclib, palbociclib, AZD-5348, AMG-935 | 0.01-20 µM  Dinaciclib IC_50_ ranging between 10 and 20 nM | - cell Proliferation Analysis - annexin V Apoptosis Assay - clonogenic Growth Assay - cell-Cycle Analysis - DiOC6 labeling and detection of mitochondrial membrane depolarization - Immunoblot - fluorescence microscopy | - no significant growth inhibition by ribociclib and palbociclib   - dinaciclib inhibited cell proliferation, induced cell-cycle arrest (G2/M checkpoint) - synergistic killing by ABT-737 with dinaciclib: apoptosis, early loss of mitochondrial transmembrane potential; the release of cytochrome c, smac/DIABLO, apoptosis-inducing factor; phosphatidylserine exposure, caspase and PARP activation - dinaciclib promoted proteasomal degradation of Mcl-1, but no altered CDK1, CDK2, CDK4, CDK6, and CDK7 expression levels, only reduction of CDK9 |
| *in vitro, in vivo*[97] | GBM cell lines U87 and U251, U87/EGFRvIII and U87/PTEN cell lines  GBM10 | ON123300 (CDK4 inhibitor), ON1231120, ON1231320, gefitinib,  TMZ | *in vitro*:  ON123300: 0.03 - 16 μmol/L  ON123300: 0.1 - 25 μmol/L  gefitinib: 0.16 - 80 μmol/L  TMZ: 10 - 2,500 μmol/L  *in vivo*:  ON123300, 5 and 25 mg/kg bw  gefitinib 200 mg/kg | - cytotoxicity and combination drug studies - flow cytometric analysis - live cell microscopy - protein sample preparation - phospho-MAPK array - immunoblot - meso Scale Discovery assay - orthotopic glioma model and dosing studies - LC/MS-MS analysis - pharmacokinetic and pharmacodynamic data | ON123300   - inhibited cell proliferation - decreased expression of pAkt, CREB, JNK - p-Erk and p-p38γ increased - inhibited phosphorylation of Akt, P70S6K, 40S ribosomal protein S6, Rb S780 - regulated p-Akt and p-Erk activities through PDGFRB and FGFR1 signaling pathways - *in vitro* combination studies: gefitinib enhanced ON123300 blockade of p-Akt, p-Ark5, and p-mTOR + decreased p-Erk levels - effects on cell-cycle progression   *In vivo*   - pharmacokinetic/pharmacodynamic studies - decreased p-Akt expression, increased p-Erk activity in brain tumors |
| *in vitro, in vivo* [98] | Patient-derived DIPG cell cultures (U-DIPG-IV: H3.1-K27M; SU-DIPG-VI/XIII-P, JHH-DIPG1, SF7761: H3.3-K27M) | JQ1, Panobinostat, OTX015, iBET762, AZD8055, THZ1, SCH772984, LDN-211904 | *in vitro*:  JQ1: 0.1 μM to 10 μM  THZ1: 12.5 nM to 250 nM  Panobinostat: 20 nM to 200 nM  *in vivo*:  THZ1: i.v. or i.p., 10 mg/kg bw, 5 days per week | - cell Viability and combinatorial drug synergy - proliferation, apoptosis, and cell cycle - lentivirus preparation and infection - immunoblot - LC-MS/MS analysis of tissue and serum - ChIP and library preparation - RNA Sequencing - Boyden chamber invasion assays - 3D migration and invasion assays - DIPG orthotopic xenograft model and drug testing | - Inhibited cell viability and tumor growth - identification of super-enhancers in DIPG - EPH receptor signaling plays a role in DIPG cell migration and invasion - Panobinostat and THZ1 disrupt super-enhancer biology |
| *in vitro, in vivo*  [159] | MCF7, ZR‐75‐1, T‐47D, A549, SK‐OV‐3, NCI‐H292, U‐87 MG, PC‐3, SNU‐398, SNU‐475, SNU‐182, OVCAR‐3, LN‐18, HS‐578T, MDA‐MB‐231 and MV‐4‐11 | SHR6390,  palbociclib,  4‐OH tamoxifen | *in vitro*:  SHR6390: IC_50_ < 800 nmol/L  *In vivo*:  37.5-150 mg/kg | - cell proliferation assay - immunoblot - cell cycle analysis - senescence β-galactosidase - in vivo model | SHR6390   - inhibits proliferation of Rb‐positive cells - induces G_1_‐phase cell cycle arrest and cellular senescence through inhibition of CDK4/6‐RB pathway - *in vivo* antitumor activity in human xenograft models - Combined SHR6390 and endocrine therapy: synergistic antitumor efficacy in ER‐positive breast tumor xenografts |
| *in vitro, in vivo*  [160] | SU-DIPG4, SU-DIPG13, HSJD-DIPG007, SF8628, SF7761, BT245 | Atuveciclib, AZD4573, PHA-767491, flavopiridol, LDC000067 | *in vitro*:  atuveciclib: 373-858 nM (depending on cell line)  AZD4573: 8-10 nM (depending on cell line)  *in vivo:*  atuveciclib, 30 mg/kg/dose  AZD4573, i.p. 20 mg/kg bw | - shRNA screening and transduction - proliferation assays - colony focus and neurosphere assay - aldehyde dehydrogenase assay - Extreme limiting dilution assay - Co-Immunoprecipitation - drug screening - transcriptome sequencing (RNA-seq) - ChIP sequencing - qPCR - immunoblot - orthotopic xenograft model - Immunohistochemistry | - AFF4 = epigenetically dependent in DMG - SEC = critical for DMG proliferation and mediates stem-like phenotype - CDK9 pharmacologic inhibition recapitulates phenotypic effects of SEC genetic depletion - H3K27M mutation perturbs epigenetic regulation of AFF4 - permissive CDK9 facilitates cell growth and impedes terminal morphogenesis - CDK9 Inhibition is effective *in vivo* |
| *in vitro, in vivo*  [63] | human long‐term cell lines (LN‐18, LN‐428, D247MG, LN‐319, A172, U87MG, T98G, LN‐308, LN‐229) and human glioma‐initiating cell lines (T‐325, T‐269, ZH‐161, S‐24, ZH‐305) | TG02 | *in vitro*:  14-73 nM  *In vivo*:  75 mg/kg bw | - transfections - viability assays and flow cytometry - transmission electron microscopy - Real‐time PCR - immunoblot - generation of TG02‐resistant GIC sublines - senescence β-galactosidase - Animal studies | - modulation of target gene expression - TG02‐induced cell death is independent of MGMT - no acquired resistance to TG02 |
| *in vitro, in vivo*  [62] | U87, T98G, LN229, HS683, LN18, and Human intestine epithelial cells (HIEC-6, CRL3266) cell lines | TG02, TMZ | *in vitro*:  TG02: 36-58 nmol/L  *in vivo*:  TG02 = 25-400 nmol/L  TMZ = 125-2000 μmol/L | - cell viability & apoptosis assay - plasmid construction/transient transfection - Immunoblot & colony formation assay - Preparation of subcellular fractions - Raman imaging - immunofluorescence & electron microscopy - flow cytometric detection of mitochondrial membrane potential - ATP determination - measurement of isolated complex I nicotinamide adenine dinucleotide hydride dehydrogenase activity - extracellular acidification rate measurement - RNA sequencing - intracranial GBM model, bioluminescence image - pharmacodynamics study | TG02  - induces cytotoxicity & apoptosis   - synergizes with TMZ, induces mitochondrial dysfunction and suppresses glycolysis - has minor effect on normal cells - survival benefit from combination treatment |
| *in vitro, in vivo*  [161] | patient-derived xenografts ((Non-) BTIC) | dinaciclib and LY2857785 | *in vitro*:  dinaciclib and LY2857785:  25-100 nM  *in vivo:*  dinaciclib 30 mg/kg bw 2 weeks (3 times per week) | - proliferation and neurosphere formation assay - vectors and lentiviral transfection - immunoblot - limiting dilution assay - Immunofluorescent staining - ChIP assay and ChIP-seq - Peak calling - Intracranial tumor model & bioluminescence imaging - RNA sequencing | - targeting NOTCH does not inhibit BTIC proliferation - BTICs preferentially express RBPJ without differential NOTCH activation - RBPJ = necessary for BTIC maintenance - NOTCH and RBPJ regulate different transcriptional programs - RBPJ binds to CDK9 to regulate transcription - CDK9 targeting decreases BTIC growth and self-renewal - RBPJ and CDK9 inform patient prognosis |
| *in vitro, in vivo*  [162] | cancer cell lines A549, U2OS, HCT116, U87MG, A375, MV-4-11, RPMI 8226, L363 | LY2857785, flavopiridol | *in vitro*:  IC_50_ values  *in vivo:*  LY2857785 i.v. bolus, 4, 8, 18 mg/kg bw, different schedules | - CDK7, CDK8, CDK9, and profiling biochemical kinase assays - RNAP II CTD phospho-Ser2 and -Ser5 multiplexing cell-based acumen assay - cell proliferation and apoptosis assays - RNAP II CTD Ser2 phosphorylation inhibition for *in vivo* target inhibition assay - Xenograft tumor and orthotopic myr-AKT/Eμ-Myc and Bcl2/Eμ-Myc leukemia models - Antitumor growth efficacy with xenograft models - patient with normal, AML, and CLL peripheral blood mononuclear cell sample analysis - immunoblot | Discovery of LY2857785:   - inhibits hematologic and solid tumor cell proliferation and induces apoptosis *in vitro* - antiproliferation profile predicts hematologic cancer cell sensitivity - strong inhibition of CTD phosphorylation and induction of cell death in AML and CLL - inhibits proliferation of normal human hematopoietic cells *in vitro* - inhibits RNAP II CTD Ser2 phosphorylation *in vivo* - demonstrates potent antitumor growth efficacy in preclinical tumor models |
| *in vitro* [[17]](https://www.ncbi.nlm.nih.gov/pubmed/29240261) | GBM cell lines U87 and LNZ308 | ribociclib, palbociclib, AZD5438, dinaciclib | ribociclib, palbociclib, seliciclib and AZD5438: 0.1-20 μmol/L; dinaciclib: 0.025-10 μmol/L | - Stable cell line generation (Bcl-xL) - annexinV apoptosis assay - DiOC6 labeling and detection of mitochondrial membrane depolarization - reactive oxygen species determination - cell cycle analysis - immunoprecipitation and immunoblot - subcellular fractionation - transient transfection - fluorescence microscopy | dinaciclib  - causes cell death   - induces mitochondrial dysfunction in Bcl-xL silenced cells - induces Bax and Bak conformational changes, release of caspase activators, - induces caspase-dependent cell death - induces ROS generation - downregulates cell cycle regulatory proteins in Bcl-xL depleted cells - promotes RAD51 and Ku80 proteolysis and exacerbates DNA damage response |
| *in vitro, in vivo*  [82] | GBM cell lines LN229, U87, U373, SF767, GBM43 and GBM14, breast cancer cell lines BT549, MDAMB231, T47D, and BAX-WT, and BAX-KO MEFs | roscovitine PIK-90 (PI3K inhibitor), and PI3K/mTOR inhibitors PI-103 and LY294002 | *in vitro*:  LY294002 (CDK1 [IC_50_: 5.8 μM (CDK1/cyclin B); 25 μM (CDK5)],  CDK2 (IC_50_: 6.6, 0.41, 5.5, 15, and 3.9 μM for CDK1/cyclin B, CDK2/cyclin A, CDK4/cyclin D, CDK5/p25, and CDK7/cyclin H  In vivo:  PIK-90, i.p., 40 mg/kg, daily roscovitine, i.p., 50 mg/kg, daily | - flow cytometry - siRNA transfection and retroviral transduction - immunoblot - immunohistochemistry - xenografts | - apoptosis induction, dependent on PI3K and mitochondrial-dependent - inhibition of CDK and PI3K induces synthetic lethality - CDK1/2 inhibitors cooperate with PI3K inhibitor to down-regulate survivin 🡪 apoptosis - CDK1/2 inhibitor + PIK-90: induce cell death in xenografts |

Supplementary Table 2B: Overview on running or recently completed clinical studies on GBM.

| Phase/Study design | Tumor entity/entities | Intervention | Treatment schedule | Status | Clinical trial.gov identifier |
| --- | --- | --- | --- | --- | --- |
| 1  Interventional | GBM | ribociclib | - ribociclib (LEE011) 600 mg/day for 8-21 days prior to surgery  - evaluation of efficacy and toxicity in patients with Rb-positive tumors  - treatment until unacceptable toxicity or disease progression | Unknown | NCT02345824 |
| Early 1  Interventional  Non-Randomized | GBM, brain tumor, recurrent brain tumor, | abemaciclib  bevacizumab | Arm 1: Abemaciclib 150 mg po bid PLUS Bevacizumab 10 mg/kg IV every 2 weeks, then continue treatments for 2 cycles  Arm 2: Abemaciclib 100 mg po bid PLUS Bevacizumab 10 mg/kg IV every 2 weeks, then continue treatments for 2 cycles | Recruiting | NCT04074785 |
| Early 1  Interventional | GBM | abemaciclib  LY3214996 | - LY3214996 400 mg QD for 6 doses and Abemaciclib 100 mg BID for 11 doses over 5.5 days prior to surgical resection - day 6: abemaciclib + LY3214996 dose 7 to 9 hours prior to craniotomy for tumor resection | Recruiting | NCT04391595 |
| 1 interventional | recurrent childhood GBM and other solid tumors | palbociclib isethionate | - palbociclib isethionate PO QD on days 1-21. Treatment repeats every 4 weeks for 26 courses in the absence of disease progression or unacceptable toxicity. | Terminated | NCT02255461 |
| Early 1  Interventional  Non-Randomized | GBM, Glioma of Brain | ribociclib  everolimus | - All cohorts: ribociclib and everolimus (3-14 patients) orally in 5 daily doses with the last dose being administered at one of 3 intervals before brain tumor resection: - Cohort 1: last ribociclib+everolimus dose 1 to 3 hours prior to craniotomy - Cohort 2: last ribociclib+everolimus dose 7 to 9 hours prior to craniotomy - Cohort 3: last ribociclib+everolimus dose 23 to 25 hours prior to craniotomy - lead-in dose escalation study: ribociclib 400 mg and everolimus 2.5 mg orally in 5 daily doses with the last dose - DLT: regimen considered safe and continue with the dose escalation phase to Level 3   Four dose escalation levels:  Level 0: ribociclib 400mg and everolimus 2.5  Level 1: ribociclib 600mg and everolimus 2.5mg  Level 2: ribociclib 600mg and everolimus 5mg  Level 3: ribociclib 600mg and everolimus 10mg | Recruiting | NCT03834740 |
| 1  Interventional  Non-Randomized  dose escalation | GBM, Glioma and other solid tumors | Gemcitabine  ribociclib  sonidegib  trametinib  filgrastim | Experimental: A: ribociclib + gemcitabine  Stratum A participants: refractory or recurrent medulloblastoma (Group 3/4) or refractory or recurrent ependymoma   - combination treatment with ribociclib and gemcitabine (± growth therapy support with filgrastim)   Experimental: B: ribociclib + trametinib  Stratum B participants: refractory or recurrent CNS diseases   - combination treatment with ribociclib and trametinib   Experimental: C: ribociclib + sonidegib  Stratum C participants: refractory or recurrent medulloblastoma (SHH-activated) >6 months off smoothened inhibitor, presence of 9q loss or PTCH1 mutant, skeletally mature   - combination treatment with ribociclib and sonidegib | Recruiting | NCT03434262 |
| 1  Interventional  Non-Randomized  Dose Escalation | Relapsed Solid Tumor Refractory Solid Tumor | abemaciclib  Irinotecan  TMZ | Arm 1: dose escalation and expansion: Abemaciclib (p.o.) + Irinotecan (i.v.) + TMZ (p.o.)  Arm 2: dose escalation: Abemaciclib + TMZ | Not yet recruiting | NCT04238819 |
| 1  Interventional  Non-Randomized | Astrocytoma, Grade III Glioblastoma | TG02  Radiation: Radiation Therapy  Temozolomide | Arm 1: TG02 + RT   - elderly patients with IDH1R132H-non mutant and MGMT promoter-unmethylated anaplastic astrocytoma or glioblastoma - groups A and B: TG02 at 200 mg on intermittent schedules in combination with either RT or TMZ. TG02 escalated to 250 mg if the dose decision criteria are met in the first cohort - group A: standard involved-field hypofractionated RT 39.9 Gy in 15 fractions of 2.66 Gy for 3 weeks   Arm 2: TG02 + TMZ   - elderly patients with IDH1R132H-non mutant and MGMT promoter-methylated anaplastic astrocytoma or glioblastoma - groups A and B: TG02 at 200 mg on intermittent schedules in combination with either RT or TMZ. TG02 to 250 mg if the dose decision criteria are met in the first cohort - group B: TMZ will be given in the standard 28-day cycle regimen (150-200 mg/m2) for 5 days   Arm 3: TG02   - patients initially diagnosed with anaplastic astrocytoma or glioblastoma at first relapse post TMZ/RT --> TMZ therapy who will receive TG02 - initial cohort in group C: TG02 alone at 250 mg on intermittent schedules, continued if feasible or decreased to 200 or 150 mg if not tolerated | Recruiting | NCT03224104 |
| 2  Interventional  Non-Randomized | GBM | Pembrolizumab  abemaciclib | Arm 1: surgery   - reoperation and evidence of CDKN2A/B or C loss and intact RB from a prior tumor sample - Pembrolizumab-prior to surgery, at predetermined dose and time point - abemaciclib: every 12 hours from the day of pembrolizumab infusion to the morning of surgery - post surgery participants   - Abemaciclib, twice daily oral at specified dose for 21 day cycle   - Pembrolizumab i.v. over 30 minutes once in 21 day cycle (3 weeks) (+/- 3 days)   Arm 2: non-surgery   - Abemaciclib, twice daily oral at specified dose for 21 day cycle   - Pembrolizumab intravenous over 30 minutes once in 21 day cycle (3 weeks) (+/- 3 days) | suspended | NCT04118036 |
| 2  Interventional  Non-Randomized | GBM | abemaciclib (LY2835219)  Procedure: Surgery | Arm 1: with surgery   - continuous twice daily dosing schedule - if re-operation required: short preoperative course of abemaciclib - tissue used to investigate the ability of abemaciclib to pass the blood brain barrier - after recovery from surgery, participants will resume abemaciclib (28 days/cycle).   Arm 2: without surgery   - abemaciclib administered on a continuous twice daily dosing schedule (28 days/cycle) | Active, not recruiting | NCT02981940 |
| 2  Interventional randomized | GBM | TMZ  neratinib  CC-115 (CC0483115)  abemaciclib (LY2835219) | Arm 1: TMZ   - daily (maximum 49 days) - TMZ p.o. daily 2-3 hours before RT and post RT for up to 6 cycles (5 days/cycle)   Arm 2: abemaciclib + TMZ   - daily RT (maximum 49 days) - TMZ p.o. daily during RT, 2-3 hours before RT - abemaciclib post RT p.o., twice daily pre-determined dose   Arm 3: CC-115   - twice daily p.o., daily RT (maximum 49 days) - CC115 twice daily post RT   Arm 4: Neratinib + TMZ   - daily RT (maximum 49 days) - TMZ p.o. daily during RT, 2-3 hours before RT - neratinib post RT, daily p.o. pre-determined dose | Recruiting | NCT02977780 |
| 1/2  Interventional  Non-Randomized | GBM, adult | APG101  Alectinib  Idasanutlin (Mdm2-antagonist)  Atezolizumab  Vismodegib  Temsirolimus  Palbociclib | Arm 1: APG101   - 800 mg i.v., weekly for 6 months or until progression - + RT (60 Gy in 2 Gy fractions) for the first 6 weeks   Arm 2: alectinib   - 600 mg p.o., twice daily for 6 months or until progression - + RT (60 Gy in 2 Gy fractions) for the first 6 weeks   Arm 3: Idasanutlin   - 100 mg until MTD, p.o., daily on five consecutive days of a 28-day cycle for 6 months or until progression - + RT (60 Gy in 2 Gy fractions) for the first 6 weeks   Arm 4: Atezolizumab   - application of 1200 mg i.v. every three weeks for 6 months or until progression - + RT (60 Gy in 2 Gy fractions) for the first 6 weeks   Arm 5: Vismodegib   - daily application of 150 mg orally for 6 months or until progression - + RT (60 Gy in 2 Gy fractions) for the first 6 weeks   Arm 6: Palbociclib   - 75/100/125 mg orally once daily on 21/28 days - + RT (60 Gy in 2 Gy fractions) for the first 6 weeks - followed by 4 weeks break (after last dose of 2nd cycle) - + maintenance therapy with palbociclib, 125 mg daily for 6 months or until progression   Arm 7: Temsirolimus   - weekly application of 25 mg i.v. for 6 months or until progression - + RT (60 Gy in 2 Gy fractions) for the first 6 weeks | Recruiting | NCT03158389 |
| 1  Interventional | Recurrent Childhood GBM and other solid tumors | palbociclib isethionate | - QD on days 1-21, treatment repeats every 4 weeks for 26 courses in the absence of disease progression or unacceptable toxicity | Terminated | NCT02255461 |
| Early 1  Interventional | GBM, meningioma | Ribociclib (LEE011) | - prior to surgical resection; p.o.5 doses of LEE011 (900 mg/d) with the final dose occurring at one of 3 following intervals before brain tumor resection:   - Cohort 1: last ribociclib dose 2-4 hours prior to craniotomy - Cohort 2: last ribociclib dose 6-8 hours prior to craniotomy - Cohort 3: last ribociclib dose 23-25 hours prior to craniotomy | Recruiting | NCT02933736 |
| 1  Interventional  Non-Randomized | GBM,  Glioma | ribociclib  everolimus | Arm 1: Dose level 1 (starting dose level) (participants </= 21yrs)   - ribociclib p.o.; daily on days 1-21 each 28 day cycle; dose calculation age dependent (>21 yrs of age 300mg daily (DIPG only); </=21 yrs of age 120 mg/m2/day)   - everolimus p.o.; daily on days 1 - 28 each 28 day cycle; dose calculation age dependent (>21yrs 2.5mg/day (DIPG only); </=21yr 1.2 mg/m2/day) BSA >/=0.75m2  Arm 2: Dose level 2 (participants </= 21yrs)   - ribociclib p.o.; daily on days 1-21 each 28 day cycle; 170 mg/m2/day - everolimus p.o.; daily on days 1 - 28 each 28 day cycle; 1.2 mg/m2/day BSA >/=0.45m2   Arm 3: Dose level 3 (participants </= 21yrs)   - ribociclib p.o.; daily on days 1-21 each 28 day cycle; 170 mg/m2/day - everolimus p.o.; daily on days 1 - 28 each 28 day cycle; 1.5 mg/m2/day BSA >/=0.45m2   Arm 4: Dose level 1 (DIPG participants > 21yrs)   - ribociclib p.o.; daily on days 1-21 each 28 day cycle; 300mg daily - everolimus p.o.; daily on days 1 - 28 each 28 day cycle; 2.5mg/day | Active, not recruiting | NCT03355794 |
| 1/2  Interventional  Randomized | Brain Tumor,  Astrocytoma,  Astroglioma,  Gliosarcoma | zotiraciclib (TG02)  TMZ | Drug: zotiraciclib (TG02)  Phase 1: Two treatment arms & several dose levels   - TMZ (125 mg/m2 x 7 days on / 7days or 50 mg/ m2 daily) with two alternate schedules (dd and mn) + Zotiraciclib (TG02) - cohort extension of both arms at each MTD: arm with a better PFS at 4 months selected for combination 🡪 Phase 2 - TMZ + Zotiraciclib (TG02) versus dd/mn TMZ alone using a Bayesian clinical trial design. - dosage for the combination arm will be derived from the MTD determined in the Phase 1   Drug: TMZ  Phase 1: TMZ with two alternate schedules (dd and mn) + Zotiraciclib (TG02)   - cohort extension of both arms at each MTD: arm with a better PFS at 4 months selected for combination 🡪 Phase 2 - TMZ + Zotiraciclib (TG02) versus dd/mn TMZ alone using a Bayesian clinical trial design | Completed | NCT02942264 |
| 1  Interventional  Non-randomised  3 +3 design | 15 participants, recurrent / progressive GBM with failed TMZ treatment | TG02 | - 150 mg, BIW in every 28d - 200 mg, BIW in every 28d - 250 mg, BIW in every 28d - TG02 capsules, p.o., on the 1st, 4th, 8th, 11th, 15th, 18th, 22nd and 25th day, every 28 days - determine DLT, MTD, ORR and c-myc expression in tumor tissue | Recruiting | NCT03904628 |
| 1  Interventional | 22 participants, recurrent or refractory malignant brain tumors (Children) | ribociclib and everolimus | - ribociclib: p.o, daily: on days 1-21 of course 1, days 2-21 of course 2 - everolimus: p.o., daily: on days 3-28 of course 1 and days 1-28 of subsequent courses - surgery: ribociclib: on days 7-10 before surgery - treatment repeats: every 28d for up to 13 courses (in absence of disease progression or unacceptable toxicity) - SD: continue receiving ribociclib and everolimus every 28d max. 13 additional courses - determine DLT, MTD, RP2D, pharmacokinetics, potential for drug-drug interactions - characterize concentrations in tumor and plasma - describe response rate - knowledge of the genomic landscape of treatment-refractory pediatric CNS tumors | Completed | NCT03387020 |
| 1/2  Interventional  Non-Randomized | 24 participants, High Grade Glioma Diffuse Intrinsic Pontine Glioma Bithalamic High Grade Glioma | ribociclib | - orally, once daily: on days 1- 21 each 28 day cycle; dose calculation age dependent (>21 yrs of age 600mg daily (DIPG); <21 yrs of age 350 mg/m^2^/day) - determine AE, patients alive at one year, PFS, pseudoprogression | Unknown | NCT02607124 |

DLT: dose limiting toxicities; i.p.: intraperitoneal; i.v.: intravenous; IMRT: Intensity Modulated Radiation Therapy; MTD: Maximum Tolerated Dose; PFS – progression-free survival; p.o.: per oral; RP2D: Recommended Phase 2 Dose; RT – radiotherapy; SD – stable disease; TMZ - Temozolomide

Supplementary Table 2B: Overview on completed clinical studies on GBM.

| Phase/  Study design/  Reference | Tumor entity/entities | Intervention | Treatment schedule | Treatment-related toxicities | Outcome | Reference |
| --- | --- | --- | --- | --- | --- | --- |
| 0 | recurrent GBM | ribociclib | - daily, 900 mg for 5 days before tumor resection - blood, tumor, and cerebrospinal fluid (CSF) samples collected - total and unbound ribociclib concentrations |  | - mean unbound ribociclib concentrations in CSF, non-enhancing, and enhancing tumor regions: 0.374 μmol/L, 0.560, and 2.152 μmol/kg, - more than 5-fold the *in vitro* IC_50_ for CDK4/6 inhibition (0.04 μmol/L) - G_1_-to-S phase suppression inferred by decreases in pRB (*P* < 0.01) and cellular proliferation (*P* < 0.05). - 6/12 patients enrolled into the pharmacokinetic/pharmacodynamic-guided expansion cohort - PFS: 9.7 weeks - upregulation of the PI3K/mTOR pathway | **31285369** |
| 1 multicenter | GBM and other solid tumors | abemaciclib | dose escalation:  3+3 design.   - two continuous administration schedules: 50, 100, 150, or 225 mg p.o. every 24 hours and 75, 100, 150, 200, or 275 mg p.o. every 12 hours - MTD: abemaciclib (150 mg Q12H or 200 mg Q12H) assessed in tumor-specific cohorts | - DLT: grade 3 fatigue | - MTD: 200 mg every 12 hours - radiographic responses in previously treated patients with breast cancer, NSCLC, and melanoma - hormone receptor–positive breast cancer: ORR 31%; 61% of patients achieved either response or stable disease lasting ≥6 months. | **27217383** |
| 1 multicenter, non-randomized, open-label, study | 224 patients, GBM and other solid tumors | abemaciclib | - doses ranging : 50 to 225 mg every 24 h and 75 to 275 mg every 12 h - pharmacokinetics: time- and dose-dependent relative bioavailability (*F* _rel_) - clinical dose-response relationship for target engagement in epidermal keratinocytes and the correlation of pRb suppression to clinical activity in phase 1 supports the dose levels of abemaciclib (150 and 200 mg q12h) |  |  | 28540640 |
| 2 | 22 patients, recurrent RB1-positive GBM | palbociclib | Surgical group and non-surgical group:   - 125 mg daily for 7 days prior surgery - after recovery: repeating schedule of 21 consecutive days of drug followed by a 7 day break off therapy (cycle length = 28 days) - in the absence of disease progression = 12 cycles, up to max of 24 cycles | - 2 patients: grade ≥ 3 AEs | - 6 patients on arm 1 and 16 patients on arm 2 - Stopped: lack of efficacy: 95% progressing within 6 months - Median PFS = 5.14 weeks (range 5 days–142 weeks) - median OS = 15.4 weeks (range 2–274 weeks) - Arm 1, 5: tissue concentrations = not sufficient for biological effect | 30151703 |

DLT: dose limiting toxicity; ORR: overall response rate; p.o.: per oral

**References**

150. Mishra, P. B., Lobo, A. S., Joshi, K. S., Rathos, M. J., Kumar, G. A., & Padigaru, M. (2013). Molecular mechanisms of anti-tumor properties of P276-00 in head and neck squamous cell carcinoma. *Journal of Translational Medicine, 11* (1). <https://doi.org/10.1186/1479-5876-11-42>.

151. Hsu, C.-L., Lui, K.-W., Chi, L.-M., Kuo, Y.-C., Chao, Y.-K., Yeh, C.-N., et al. (2018). Integrated genomic analyses in PDX model reveal a cyclin-dependent kinase inhibitor Palbociclib as a novel candidate drug for nasopharyngeal carcinoma. *Journal of Experimental & Clinical Cancer Research : CR, 37* (1), 233.

<https://doi.org/10.1186/s13046-018-0873-5>.

152. Seront, E., Schmitz, S., Papier, M., Van Maanen, A., Henry, S., Lonchay, C., et al. (2019). Phase 1 study evaluating the association of the cyclin-dependent kinase 4/6 inhibitor ribociclib and cetuximab in recurrent/metastatic p16-negative squamous cell carcinoma of the head and neck. *Frontiers in Oncology, 9*(MAR). <https://doi.org/10.3389/fonc.2019.00155>.

153. Rathkopf, D., Dickson, M. A., Feldman, D. R., Carvajal, R. D., Shah, M. A., Wu, N., et al. (2009). Phase I study of flavopiridol with oxaliplatin and fluorouracil/leucovorin in advanced solid tumors. *Clinical Cancer Research, 15*(23), 7405–7411. <https://doi.org/10.1158/1078-0432.CCR-09-1502>.

154. Dickson, M. A., Rathkopf, D. E., Carvajal, R. D., Grant, S., Roberts, J. D., Reid, J. M., et al. (2011). A phase I pharmacokinetic study of pulse-dose vorinostat with flavopiridol in solid tumors. *Investigational New Drugs, 29*(5), 1004–1012. <https://doi.org/10.1007/s10637-010-9447-x>.

155. Zhai, S., Sausville, E. A., Senderowicz, A. M., Ando, Y., Headlee, D., Messmann, R. A., Arbuck, S., Murgo, A. J., Melillo, G., Fuse, E., & Figg, W. D. (2003). Clinical pharmacology and pharmacogenetics of flavopiridol 1-h i.v. infusion in patients with refractory neoplasms. *Anti-Cancer Drugs, 14*(2), 125–135.

<https://doi.org/10.1097/00001813-200302000-00006>.

156. Greenall, S. A., Lim, Y. C., Mitchell, C. B., Ensbey, K. S., Stringer, B. W., Wilding, A. L., et al. (2017). Cyclin-dependent kinase 7 is a therapeutic target in high-grade glioma. *Oncogenesis, 6*(5). <https://doi.org/10.1038/oncsis.2017.33>.

157. Long, F., He, Y., Fu, H., Li, Y., Bao, X., Wang, Q., et al. (2019). Preclinical characterization of SHR6390, a novel CDK 4/6 inhibitor, in vitro and in human tumor xenograft models. *Cancer Science, 110*(4), 1420–1430. <https://doi.org/10.1111/cas.13957>.

158. Dahl, N. A., Danis, E., Balakrishnan, I., Wang, D., Pierce, A., Walker, F. M., et al. (2020). Super elongation complex as a targetable dependency in diffuse midline glioma. *Cell Reports, 31*(1). <https://doi.org/10.1016/j.celrep.2020.03.049>.

159. Xie, Q., Wu, Q., Kim, L., Miller, T. E., Liau, B. B., Mack, S. C., et al. (2016). RBPJ maintains brain tumor-initiating cells through CDK9-mediated transcriptional elongation. *Journal of Clinical Investigation, 126* (7), 2757–2772. <https://doi.org/10.1172/JCI86114>.

160. Yin, T., Lallena, M. J., Kreklau, E. L., Fales, K. R., Carballares, S., Torrres, R., et al. (2014). A novel CDK9 inhibitor shows potent antitumor efficacy in preclinical hematologic tumor models. Molecular Cancer Therapeutics, 13(6), 1442–1456. https://doi.org/10.1158/1535-7163.MCT-13-0849.
